# Entwicklung einer Entscheidungshilfe für partizipative Vorausplanungen für Menschen mit Demenz und deren Angehörige

**DOI:** 10.1007/s00115-020-00911-2

**Published:** 2020-04-28

**Authors:** Katharina Bronner, Lea Bodner, Ralf J. Jox, Georg Marckmann, Janine Diehl-Schmid, Johannes Hamann

**Affiliations:** 1 Klinik und Poliklinik für Psychiatrie und Psychotherapie, Klinikum rechts der Isar, Technische Universität München, Ismaninger Straße 22, 81675 München, Deutschland; 2 Institut für Ethik, Geschichte und Theorie der Medizin, Ludwig-Maximilians-Universität München, München, Deutschland

**Keywords:** Patientenverfügung, Entscheidungshilfe, Partizipative Entscheidungsfindung, Alzheimer Erkrankung, Patientenautonomie, Alzheimer´s Disease, Advance Care Planning, Shared decision Making, Decision Aid, Patient Autonomy

## Abstract

**Hintergrund:**

Eine Demenzdiagnose konfrontiert Betroffene mit vielen gesundheitlichen und sozialen Entscheidungen. Aufgrund der Progression der Demenz ist für eine aktive Teilnahme am Entscheidungsprozess eine rechtzeitige Auseinandersetzung mit diesen Themen ratsam. Eine professionelle Unterstützung kann dabei helfen, frühzeitig gemäß den eigenen Wünschen und Möglichkeiten vorauszuplanen.

**Material und Methoden:**

In einem mehrstufigen Prozess wurde eine Entscheidungshilfe basierend auf „advance care planning“ und „shared decision making“ entwickelt. Der Prototyp wurde an 8 Patient-Angehörigen-Dyaden aus einer Spezialambulanz für Früherkennung vorgetestet und für deren Bedürfnisse bestmöglich angepasst. In einer Pilotstudie wurde anschließend die Anwendbarkeit der Entscheidungshilfe bei weiteren 19 Patient-Angehörigen-Dyaden (Diagnose einer Alzheimer-Demenz bzw. gemischte Form; MMSE (Mini-Mental-State-Test-Summenwert) >20 und <27) mit ausgebildeten Gesprächsbegleitern als Intervention getestet.

**Ergebnis:**

Das Ergebnis ist eine schriftliche Entscheidungshilfe für Menschen mit Demenz im Frühstadium und deren Angehörige, welche den Entscheidungsprozess bei wichtigen Themen (Vorsorgevollmacht, Patientenverfügung, Wohnen, Autofahren) unterstützt. Erste Ergebnisse weisen auf eine gute Akzeptanz und Handhabung hin. Patienten und Angehörige beschäftigten sich in hohem Maße mit den Themen und sprachen ihnen hohe Relevanz zu.

**Diskussion:**

Trotz positiver Rückmeldung der Teilnehmer hinsichtlich Akzeptanz und Anwendbarkeit gab es größere Schwierigkeiten bei der Rekrutierung. Perspektivisch könnte der systematisierte Einsatz einer Entscheidungshilfe als Teil der Routineversorgung dazu beitragen, Entscheidungsprozesse dieser Patientengruppe zu unterstützen.

**Zusatzmaterial online:**

Die Onlineversion dieses Beitrags (10.1007/s00115-020-00911-2) enthält weitere Infomaterialien. Beitrag und Zusatzmaterial stehen Ihnen auf www.springermedizin.de zur Verfügung. Bitte geben Sie dort den Beitragstitel in die Suche ein, das Zusatzmaterial finden Sie beim Beitrag unter „Ergänzende Inhalte“.

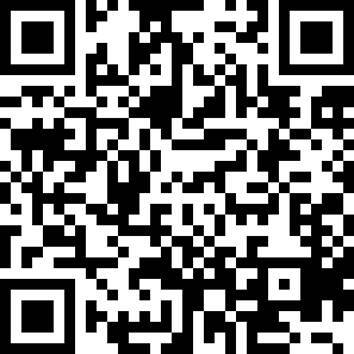

## Abstract (en)

**Background:**

Patients with a diagnosis of dementia face various important social and health-related decisions. Due to the progression of the disease it seems crucial that patients try to deal with these decisions early in the course of the disease to have the opportunity to make decisions autonomously. Professional support can help to plan in advance according to the wishes and possibilities in an effective and individualized manner.

**Material and methods:**

The instrument was developed in a multiphase process based on advance care planning and shared decision-making. The prototype was pretested on 8 patient-relative dyads from a special outpatient department for early recognition and adapted as best as possible to their needs. Subsequently, in a pilot study the applicability of the decision aid was tested as an intervention in a further 19 patient-relative dyads with trained conversion attendants (diagnosis of Alzheimer’s dementia or mixed form; mini mental state examination, MMSE (Mini-Mental-State-Test-Summenwert) >20 and <27).

**Results:**

The result was a written decision-making aid for people with early stage dementia and their relatives, which supports the decision-making process (health care proxy, advance directive, living and care, driving ability). The first results showed good acceptance and handling. Patients and relatives dealt with the individual topics to a high degree and found them to be highly relevant.

**Conclusion:**

Despite positive feedback from the participants with respect to acceptance and applicability, there were major difficulties in recruiting. In the future, the systematic use of decision support as part of routine care could help to support the decision-making process in this patient group.

## Hintergrund

### Demenz und Entscheidungsfähigkeit

Im Verlauf einer Demenz kommt es zu einer zunehmenden Einschränkung der Alltagskompetenz und in den späteren Stadien zu einer Pflegebedürftigkeit der Erkrankten [2, 21]. Grundlegende gesundheitliche und soziale Themen, die den Erkrankten und ihren Angehörigen oft Richtungsentscheidungen abverlangen, haben deswegen große Bedeutung [22]. Aus ethischer Sicht und nach Forderung verschiedener Patientenorganisationen sollten Menschen mit einer Demenzerkrankung so weit wie möglich in diese Entscheidungen einbezogen werden [13]. Die Belastung, sich mit schwierigen Entscheidungen zu beschäftigen [10, 33], führt nicht selten dazu, dass Entscheidungen bis zum Verlust der Einwilligungs- und Geschäftsfähigkeit [28] aufgeschoben werden. Diese Entscheidungen müssen dann von den rechtlichen Vertretern, meist Angehörigen, getroffen werden, was diese erheblich belastet [35] und die Gefahr birgt, dass die Wünsche der Menschen mit Demenz nicht respektiert werden [30].

### Frühe Diagnosestellung nutzen

Die Diagnosestellung einer Demenz erfolgt immer früher im Krankheitsverlauf, nach den neuesten Diagnosekriterien bei der Alzheimer-Krankheit sogar unabhängig von klinischen Symptomen [20]. Diese Entwicklung kann dafür genutzt werden, Menschen mit Demenz zu einem Zeitpunkt, an dem ihre kognitiven Fähigkeiten und damit ihre Einwilligungs- und Geschäftsfähigkeit noch erhalten sind, größtmögliche Selbstbestimmung bezüglich der ärztlichen Behandlung, der Lebensgestaltung und der künftigen Versorgung zu ermöglichen [9]. Es hat sich gezeigt, dass in frühen Stadien der Erkrankung sowohl die Entscheidungsfähigkeit in wichtigen Bereichen noch weitgehend erhalten ist, als auch Interesse an sozialen und medizinischen Entscheidungen besteht [14, 27].

### „Advance care planning“

Das Modell des „advance care planning“ (ACP) strebt u. a. die Realisierung wirksamer Patientenverfügungen an [7, 23]. Hierbei erhalten Menschen, insbesondere mit chronischen oder lebenslimitierenden Erkrankungen oder im hohen Alter im Rahmen eines professionell begleiteten Gesprächsprozesses die Möglichkeit, individuelle Präferenzen für zukünftige medizinische Behandlungen bei Verlust der Einwilligungsfähigkeit zu entwickeln und aussagekräftig zu dokumentieren [18].

Mit ACP werden die Wünsche zur Behandlung am Lebensende besser respektiert und umgesetzt [8, 26], was unnötige und unerwünschte Krankenhauseinweisungen verhindern kann [37] bei verbesserter Lebensqualität in der letzten Lebensphase [6, 8].

Auch für Menschen mit einer demenziellen Erkrankung wird ACP als sinnvolles Konzept erachtet [15, 34]. Erste Studien weisen auf positive Effekte von ACP für Menschen mit Demenz hin [11]. Allerdings gibt es noch kein etabliertes ACP-Programm, das spezifisch auf Menschen mit einer Demenz im Frühstadium fokussiert [3, 28].

### „Shared decision making“ und „decision aids“

Das Modell des „shared decision making“ (SDM) [12] zielt auf die Einbeziehung von Patienten in aktuelle medizinische Entscheidungen ab. Häufig werden als Intervention sog. Entscheidungshilfen („decision aids“) eingesetzt [31]. Es handelt sich dabei um Medien in verschiedenen Formen (z. B. Broschüren, CDs, Videos etc.), die Patienten evidenzbasiert und in verständlicher Sprache relevante Informationen zu verschiedenen Optionen nahebringen und bei ihrer Präferenzbildung unterstützen, um als informierte und kompetente Personen medizinische Entscheidungen zu treffen.

## Zielsetzung

Im Rahmen der Studie „Partizipative Planung gesundheitlicher und sozialer Entscheidungen nach Diagnosestellung einer Alzheimer-Demenz“ (DFG-Projekt HA 7115/1-1) verfolgten wir zunächst das Ziel, eine komplexe Intervention in einem mehrstufigen Prozess zu entwickeln, die in einem zweiten Schritt im Hinblick auf Akzeptanz und Anwendbarkeit pilotiert wird. Die Intervention, die sich an Menschen mit einer Alzheimer-Demenz (AD) und deren Angehörige richtet, kombiniert Bestandteile des ACP und SDM. Sie besteht aus einer schriftlichen Entscheidungshilfe (SDM-Anteil; siehe JETZT-Broschüre, Electronic Supplementary Material) und zwei persönlichen Beratungsgesprächen mit dazugehörigem Gesprächsleitfaden (ACP-Anteil), die miteinander interagieren. Unsere Hypothese ist, dass diese Intervention Menschen mit beginnender AD auf relevante Vorsorgethemen aufmerksam macht und diese dann mehr als bisher psychosoziale Entscheidungen rechtzeitig planen und umsetzen.

## Methode

### Entwicklung der Intervention

#### Recherche und Vorstudie

Eine wissenschaftliche Literaturrecherche (in PubMed) mit den Suchbegriffen „advance care planning“, „shared decision making“, „decision aid“ in Kombination mit „dementia“, und „cognitive impairment“ zeigte, dass bisher keine empirischen Studien zu ACP im Frühstadium einer Demenz publiziert wurden. Der Einsatz von ACP bei älteren (nichtdementen) Personen verbessert die Qualität von Vorausverfügungen [17] und verringert die depressive und ängstliche Symptomatik [8]. In einer Übersichtsarbeit zu „decision aids“ für ACP konnte gezeigt werden, dass durch Anwendung der Interventionen das Wissen der Patienten über ACP verbessert und die Anzahl an erstellten Patientenverfügungen gesteigert werden konnten [1].

Zudem konnte auf Ergebnisse einer qualitativen Vorstudie zurückgegriffen werden, in der von Patienten, Angehörigen und Ärzten Entscheidungsthemen wie medizinische Behandlung, Unterstützung im Alltag und rechtliche Belange unmittelbar nach der Diagnose einer Demenz im Frühstadium als besonders bedeutend identifiziert wurden [4], die auch in Leitlinien empfohlen werden [29]. Zusätzlich zeigte sich, dass Menschen mit Demenz und Angehörige nach der Diagnosestellung Unterstützung brauchen, um die Relevanz der Themen, die zur Entscheidung anstehen, zu erkennen [4]. Dies deckt sich mit den Ergebnissen einer Studie zu Hinderungsgründen für ACP bei Menschen mit Demenz [16].

#### Entwicklung des Prototyps (Entscheidungshilfe und Gesprächsleitfaden)

Für die Entscheidungshilfe wurden antizipierbare Themen von besonderer Relevanz und guter Planbarkeit ausgewählt: Vorsorgevollmacht, Patientenverfügung, Wohnen, Autofahren und Nachlass. Die Themen Medikamente und andere nichtmedikamentöse Therapien wurden bewusst nicht miteinbezogen, da diese meist unmittelbar nach der Diagnosestellung besprochen werden und demnach keine Vorausplanung darstellen.

Die Struktur der Entscheidungshilfe orientiert sich am Aufbau von „decision aids“. Für jedes Thema werden die Leser nach einem Informationsteil zu den verschiedenen Optionen strukturiert angeleitet, Präferenzen zu bilden und sich danach für eine Option zu entscheiden. Im Unterschied zu den klassischen „decision aids“ berücksichtigten wir die kognitiven Einschränkungen der Menschen mit Demenz, die fehlende Evidenzlage bei vielen sozialen Entscheidungen und die Einbeziehung der Angehörigen in den Entscheidungsprozess als Besonderheiten.

Für eine einfache Lesbarkeit wurde darauf geachtet, die Texte knapp zu halten, kurze und leicht verständliche Sätze zu verwenden, eine übersichtliche Seitengestaltung einzuhalten, den Inhalt durch besonders hervorgehobene Textteile, Kästen mit Zusammenfassungen und Bilder zu gliedern und auf eine einfache Sprache zu prüfen.

Die Qualitätskriterien zur Entwicklung von „decision aids“ ([19]; u. a. Beschreibung verschiedener Optionen und deren Folgen, Darstellung von Wahrscheinlichkeiten von Behandlungsoutcomes, Unterstützung bei der Auseinandersetzung mit den Informationen und bei der Entwicklung von Präferenzen) wurden für die Zielgruppe adaptiert. Statt einer Darstellung der wissenschaftlichen Evidenz wurden die verschiedenen Optionen durch eine Gegenüberstellung von Beweggründen und Zitaten für und gegen die Entscheidung verdeutlicht.

Eine Einbeziehung der Angehörigen bei der gemeinsamen Entscheidungsfindung ist unabdingbar, da Menschen mit Demenz überwiegend zu Hause von Familienmitgliedern betreut werden und diese von den dargestellten Themen meist unmittelbar selbst betroffen sind [5]. Dabei ist der Wunsch nach Autonomie von Menschen mit Demenz, für sich gute Entscheidungen zu treffen [14], zu beachten, da Angehörige, sogar im frühen Erkrankungsstadium, die Entscheidungen für die Betroffenen übernehmen [24].

Die Entscheidungshilfe gliedert sich wie folgt: Eingangs findet sich ein klar strukturiertes Inhaltsverzeichnis und ein einführendes Kapitel. Der Aufbau der einzelnen Themen wird jeweils identisch vorgenommen mit einer Eingangsfrage zu Beginn eines jeden Themenabschnitts, die beide Optionen vorstellt (z. B. „Möchte ich eine Vorsorgevollmacht erteilen?“), gefolgt von allgemeinen Informationen in Form von Antworten auf „Häufig gestellte Fragen“ zu dem jeweiligen Thema (z. B. „Was versteht man unter einer Vorsorgevollmacht?“). Die wichtigsten Aussagen werden anschließend unter der Überschrift „Das Wichtigste in Kürze“ noch einmal zusammengefasst. Am Ende jeden Kapitels werden Informationen zum weiteren Prozedere der Vorausplanung gegeben sowie wichtige Anlaufstellen und Adressen genannt.

**Figure 1:**
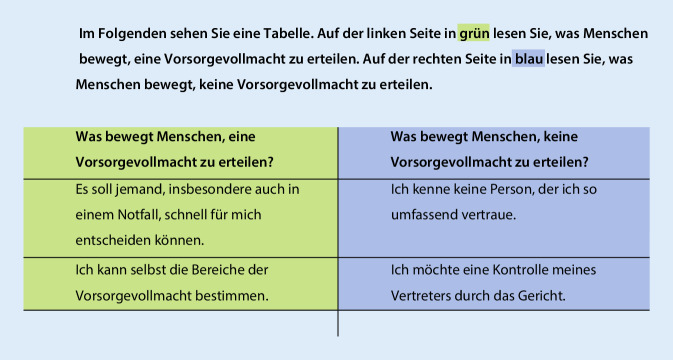

**Figure 2:**
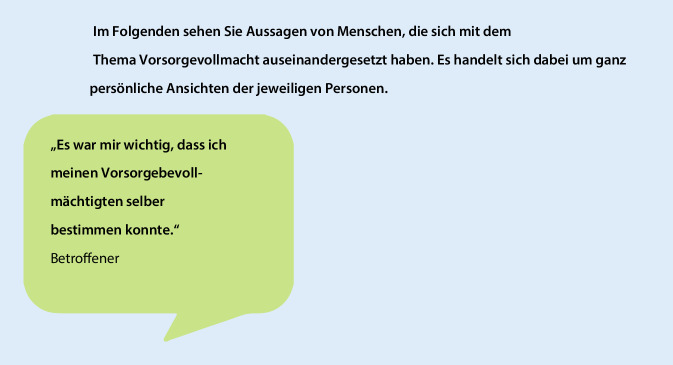

Zur Präferenzbildung werden, gestützt auf die Literatur und Erfahrungen im Expertengremium, formal und sprachlich gleichrangige Gegenüberstellungen von verschiedenen Beweggründen von Menschen mit Demenz, warum sie zu den jeweiligen Themen eine Vorsorge planen bzw. nicht planen (Abb. 1) und fiktive Zitate, die persönliche Erfahrungen von Menschen mit Demenz und deren Angehörige mit dem Thema ausdrücken (Abb. 2), verwendet. Es schließt sich ein interaktiver Teil an, in dem Patienten ihre Präferenz zu mehreren Aussagen der jeweiligen Themengebiete und zur aktuellen Planungssituation in einer abgestuften Skala angeben können (z. B. „Ich möchte die Vertrauensperson und die Bereiche der Vorsorgevollmacht selber bestimmen.“). Auch die Angehörigen werden gesondert bei jedem Thema gefragt, ob der Mensch mit Demenz eine Vorausplanung in Angriff nehmen soll oder nicht.

Parallel wurde ein Gesprächsleitfaden für die Gesprächsbegleiter entworfen, der zwei Beratungsgespräche im Abstand von einem Monat und ein Zwischentelefonat umfasst. Fester Bestandteil der Gespräche ist die Anwendung der schriftlichen Entscheidungshilfe. Menschen mit Demenz werden zusammen mit ihren Angehörigen in den beiden Gesprächen von einem Gesprächsbegleiter auf die anstehenden Entscheidungen vorbereitet. Um die Beratungsgespräche gemäß den Qualitätsstandards des ACP durchzuführen, nahmen die Gesprächsbegleiter an einer Schulung für ACP teil, die nach den Vorgaben der DiV-BVP erfolgte (bestehend aus einer mehrtägigen modularen Präsenzschulung und einem anschließend zu absolvierenden Praxisteil).

Jedes Gespräch beginnt mit einer kurzen Erklärung des organisatorischen Ablaufs und endet mit einer Anweisung zum Prozedere bis zum nächsten Termin. Im ersten Beratungsgespräch wird die Anwendung der Entscheidungshilfe anhand eines von den Teilnehmern selbst gewählten Themas exemplarisch erklärt und klare Handlungsempfehlungen zur Bearbeitung gegeben, indem das Thema zusammen bearbeitet wird. Es wird angeregt, dass der Patient (wenn möglich mit Angehörigen) bis zum nächsten Beratungsgespräch alle Themen der Entscheidungshilfe bearbeitet. Im Zwischentelefonat mit den Teilnehmern werden der Stand der Bearbeitung sowie die Themen für das zweite Beratungsgespräch erfragt und mögliche Fragen beantwortet. Im zweiten Gespräch werden weitere Themen vertieft und Fragen beantwortet. Auf Wunsch wird die Anwendung der Entscheidungshilfe ein weiteres Mal an einem priorisierten Thema erklärt.

### Überarbeitung des Prototyps

#### Expertengremium

Das Expertengremium zur Überprüfung des Prototyps bestand aus 14 Fachleuten (siehe Danksagung) aus verschiedenen Bereichen der Demenzarbeit sowie je einem Patienten- und Angehörigenvertreter. Die Expertenauswahl stützte sich dabei auf Personen, die im Großraum München über viele Jahre hinweg auf verantwortlichen Positionen im Demenzbereich gewirkt haben und über ein spezifisches Handlungs- und Erfahrungswissen verfügen. Auf bestehende Kooperationen des Zentrums für kognitive Störungen der TU München konnte dabei zurückgegriffen werden. Eine Konsensfindung wurde mit dem Verfahren des nominalen Gruppenprozesses angestrebt. Dazu wurden vor dem Treffen die Prototypen der Entscheidungshilfe und des Gesprächsleitfadens an die Teilnehmer verschickt mit der Bitte, vorab Anmerkungen schriftlich in den Texten einzufügen. Für das Expertentreffen wurden alle Kommentare gesammelt, zusammengefasst, strukturiert und im Plenum mit allen Teilnehmern diskutiert. Für alle Themen konnte dabei ein Konsens erreicht werden.

#### Überarbeitung

Nach Auswertung der Ergebnisse des Expertengremiums wurde der Prototyp modifiziert. Die Entscheidungshilfe erhielt die erweiterte Bezeichnung „Orientierungs- und Entscheidungshilfe“. Als Titel wurde ein kurzes, aufforderndes Akronym gewählt („JETZT – Jetzt Entscheidungen für die Zukunft treffen“). Es wurden vier Themen der Vorausplanung festgelegt: Vorsorgevollmacht (VV), Patientenverfügung (PV), Wohnen, Autofahren und Fahrtauglichkeit. Um den Umfang der Entscheidungshilfe zu verringern, wurde das Thema Nachlass in das rein informative Kapitel „Weitere Vorausplanungen und Entscheidungen“ integriert. Die Themen wurden inhaltlich gegliedert (rechtliche, gefolgt von den sozialen Themen) und nicht – wie anfänglich – nach der chronologischen Reihenfolge, in der die Themen im Verlauf der Demenzerkrankung relevant werden. Grund dafür war, dass die Themen VV und PV für die Anwender als zusammenhängende Einheit angesehen und oft parallel bearbeitet wurden. Das Thema „Wohnen“ wurde auf „Wohnen und Versorgung“ erweitert, da Demenzkranke überwiegend im häuslichen Umfeld gepflegt werden [32]. Die Bezeichnung „Patient“ wurde aus Sorge vor Stigmatisierung und v. a. auf Wunsch des Patientenvertreters durch die neutralere Bezeichnung „Mensch mit Demenz“ ersetzt. Weiterhin wurden die Texte im Sinne des SDM in einem weniger direktiven Ton verfasst, explizit davon ausgenommen das Thema Autofahren und Fahrtauglichkeit, da die Konsequenzen bei nicht mehr gegebener Fahreignung eine erhebliche Fremdgefährdung mit sich bringen. Deswegen wurde hier deutlich auf die gesetzliche Verpflichtung der Sicherstellung der eigenen Verkehrstauglichkeit aufmerksam gemacht.

Nach Fertigstellung des Textes wurde die Entscheidungshilfe grafisch überarbeitet. Zur besseren Orientierung wurden die verschiedenen Themen mit unterschiedlichen Farben gekennzeichnet. Zusätzlich verfügt jedes Thema zur einfacheren Wiedererkennung über ein eigenes Symbol auf jeder Seite des Kapitels. Für eine bestmögliche Lesbarkeit wurde eine Schrift mit Serifen gewählt und auf eine entsprechende Schriftgröße, einem ausreichenden Zeilen- und Wortabstand und auf einen deutlichen Schriftkontrast im Fließtext als auch bei farbigem Hintergrund geachtet [36].

Um die Inhalte der Entscheidungshilfe in möglichst einfacher und leichter Sprache zu formulieren, wurde auf geläufige Wörter, wenig Fachbegriffe und verständliche und kurze Sätze geachtet. Zusätzlich wurde die Entscheidungshilfe von Mitarbeitern der Organisation „Was hab ich?“, die sich mit der Übersetzung ärztlicher Befunde in leichte Sprache befasst, gegengelesen und redigiert.

#### Vortestung

Der modifizierte Prototyp wurde mit 8 Dyaden (Patient und Angehöriger) vorgetestet, die die Einschlusskriterien (Diagnose einer Alzheimer-Demenz oder Alzheimer/vaskulär gemischte Form; MMSE (Mini-Mental-State-Test-Summenwert) >20 und <27 oder CDR = 1; rekrutierter Angehöriger in Versorgung involviert; Dyade über Diagnose aufgeklärt) für die nachfolgende Studie erfüllten, und eine endgültige Version für die Pilotstudie erstellt. Hierbei handelte es sich um ambulante Patienten am Zentrum für kognitive Störungen der Klinik für Psychiatrie und Psychotherapie, Technische Universität München. Mithilfe der Methode des lauten Denkens [25] wurden die Benutzerfreundlichkeit, die Verständlichkeit und die inhaltliche Vollständigkeit der Themen überprüft.

## Ergebnis

Die endgültige Version (siehe JETZT-Broschüre, Electronic Supplementary Material) wurde im Rahmen einer Pilotstudie eingesetzt. Primäres Ziel der Studie ist die Evaluation der Anwendbarkeit und Akzeptanz der Intervention sowie die Generierung und Überprüfung möglicher, aussagekräftiger Outcomeparameter. Mögliche Effekte auf das Entscheidungsverhalten von Nutzern (Menschen mit Demenz und ihre Angehörige) als auch deren emotionale Reaktionen sollen abgeschätzt werden.

Insgesamt wurden 26 Dyaden rekrutiert und die Intervention mit 19 Patienten-Angehörigen-Dyaden durchgeführt (Tab. 1). Sieben Dyaden mussten aus der Studie herausgenommen werden. Gründe dafür waren u. a. akute schwere somatische Erkrankungen, die eine weitere Teilnahme ausschlossen, massive Ehekonflikte, die keine konstruktiven Gespräche zuließen, oder auch schwere kognitive Beeinträchtigungen der Angehörigen, die sich erst im Verlauf der Studie herauskristallisierten. Die 5 Studientermine (1 Einschlusstermin, 2 Beratungsgespräche, 2 Katamnesetermine) fanden auf Wunsch der Teilnehmer fast ausschließlich in deren Privatwohnungen im Großraum München statt. Erste Ergebnisse der Pilotstudie zeigen eine hohe Akzeptanz und unkomplizierte Handhabung der Entscheidungshilfe. Gefragt wurden die Teilnehmer nach ihrer Einschätzung der Einfachheit der Anwendung, der Verständlichkeit, des Informationsgehaltes und des eigenen Nutzens. Diese Daten wurden mithilfe einer 5‑stufigen Likert-Skala erhoben, in der die Teilnehmer ihre Zustimmung ausdrücken sollten. So attestierten im Durchschnitt die Patienten mit einem Mittelwert von 3,24 (neutraler Skalenmittelwert 2,0 SD ±0,83) der Entscheidungshilfe, dass diese gut verständlich sei. Eine ungefähr genauso hohe Zustimmung gab es zum Informationsgehalt (MW 3,24; SD ±0,66) bzw. dass die Entscheidungshilfe für sie von Nutzen sei (MW 3,35; SD ±0,66). Nur die Beurteilung zur Einfachheit der Anwendung fiel mit einem Mittelwert von 3,12 (SD ±0,92) etwas geringer aus. Bei den Angehörigen fiel die Zustimmung in allen 4 Kategorien mit Mittelwerten um 3,60 generell etwas höher aus (Tab. 2). Der T‑Test gegen den neutralen Skalenmittelwert war bei Patienten und Angehörigen für alle Items signifikant mit *p* < 0,0001 (Tab. 2).

**Table 1:** 

	MW (SD)	Range
**Patienten (*n*** **=** **19)**		
*Alter (Jahre)*	71,9 (±8,8)	54–86
*Geschlecht (m/w) n (%)*	11 (58)/8 (42)	–
*MMSE*	24,5 (±2,0)	20–27
*Diagnose ICD-10 n (%)*		
AD früh F00.0	5 (26,3)	–
AD spät F00.1	13 (68,4)	–
AD gemischt F00.2	1 (5,3)	–
*Zeitdauer seit Symptombeginn (Jahre)*	2,8 (±1,6)	1–6
*Zeitdauer seit Diagnosestellung (Monate)*	10,4 (±26,9)	1–120
**Angehörige (*n*** **=** **19)**		
*Alter (Jahre)*	67,8 (±11,0)	49–88
*Geschlecht (m/w) n (%)*	8 (42)/11 (58)	–
*Verwandtschaftsgrad zum Patient n (%)*		
Ehe‑/Lebenspartner	18 (95)	–
Kind	1 (5)	–

*AD* Alzheimer-Demenz, *MW* Mittelwert, *SD* Standardabweichung

**Table 2:** 

	Patient			Angehöriger		
	MW	SD	Sig^a^	MW	SD	Sig^a^
Leichte Anwendung	*3,12*	±0,92	*p* < 0,0001	3,63	±0,59	*p* < 0,0001
Gute Verständlichkeit	3,24	±0,83	*p* < 0,0001	3,68	±0,58	*p* < 0,0001
Ausreichende Informationen	3,24	±0,66	*p* < 0,0001	3,53	±0,61	*p* < 0,0001
Hoher Nutzen	3,35	±0,70	*p* < 0,0001	3,53	±0,90	*p* < 0,0001

*MW* Mittelwert, *SD* Standardabweichung, *Sig* Signifikanz

^a^T‑Test gegen den „neutralen“ Skalenmittelwert

Die Teilnehmer wurden außerdem gefragt, welche Themen sie nach Aushändigung der Entscheidungshilfe bis zum zweiten Beratungsgespräch bearbeitet hatten. Sowohl ca. 90 % der Patienten als auch der Angehörigen bearbeiteten die Hauptthemen VV, PV und Auto, wobei sich beide Parteien mit dem Thema Wohnen mit ca. 80 % etwas weniger oft beschäftigten. Das zusätzliche rein informative Kapitel „Weitere Vorausplanungen und Entscheidungen“ spielte bei beiden Parteien mit ca. 22 % eine untergeordnete Rolle (Tab. 3).

**Table 3:** 

	Thema bearbeitet *n* (%)		Thema wichtig *n* (%)	
	Patient	Angehöriger	Patient	Angehöriger
VV	16 (80)	18 (90)	14 (70)	11 (55)
PV	16 (80)	18 (90)	15 (75)	12 (60)
Wohnen	14 (70)	15 (75)	15 (75)	13 (65)
Auto	15 (75)	17 (85)	9 (45)	14 (70)
Weitere Planungen	4 (20)	4 (20)	2 (10)	4 (20)

*VV* Vorsorgevollmacht, *PV* Patientenverfügung

Zwischen 80 und 90 % der Patienten empfanden die Themen VV, PV und Wohnen als wichtig. Das Thema Auto wurde mit 53 % am seltensten genannt. Auf der Angehörigenseite erreichten die Themen VV, PV und Wohnen mit 58–68 % etwas niedrigere Zustimmungsraten. Nur das Thema Auto wurde mit 73 % als wichtiges Thema genannt. Mit 12 % (Patienten) bzw. 22 % (Angehörige) Nennungen wird dem Kapitel „Weitere Vorausplanungen“ von den Teilnehmern keine hohe persönliche Relevanz zugeschrieben (Tab. 3).

## Ausblick

Weitere geplante Auswertungen der Pilotstudie befassen sich mit dem in zwei Follow-up-Terminen nach den beiden Beratungsgesprächen erhobenen Wohlbefinden und der Lebenszufriedenheit der Teilnehmer sowie der Belastung der Angehörigen. Überdies werden die tatsächlich getroffenen Entscheidungen der Vorausplanungen mit dem Stand vor der Intervention verglichen. Dazu wurde ein strukturierter Fragebogen entworfen. Die qualitative Datenerhebung wurde mit halbstandardisierten Interviews durchgeführt. Die 4 Leitfragen bezogen sich auf die (1) Gründe und Motive, warum Entscheidungen vorgenommen oder abgelehnt wurden, (2) Faktoren, die die Entscheidungen erleichtert bzw. erschwert haben, (3) Bewertung der Entscheidungshilfe und (4) auf die emotionale Reaktion der Dyaden bei Beschäftigung mit den Themen der Entscheidungshilfe. Eine spätere randomisiert-kontrollierte Interventionsstudie könnte die Effektivität bei der Umsetzung von Entscheidungen bei Anwendern der Orientierungs- und Entscheidungshilfe mit begleitenden Beratungsgesprächen evaluieren und mit einer Kontrollgruppe ohne Intervention vergleichen. Eine zukünftige Implementierung einer Kombination der Orientierungs- und Entscheidungshilfe mit dazugehörigen begleitenden Beratungsgesprächen in der Regelversorgung wäre wünschenswert, z. B. als Angebot in Hausarztpraxen oder Senioren- bzw. Pflegeheimen.

Limitationen, die sich bereits in unserer Pilotstudie gezeigt haben (enges Zeitfenster zwischen Diagnosestellung und schwereren kognitiven Defiziten, Schwierigkeit des Zugangs zu Patienten und Angehörigen), müssen dabei adressiert werden. So wäre mit Versionen in anderen Sprachen die Entscheidungshilfe auch noch für einen größeren Personenkreis (Migrationshintergrund) verfügbar. Da die Gesprächsbegleiter nur dazu anregen, sich mit den Themen und Entscheidungen auseinanderzusetzen und keine Gespräche im Sinne des „advance care planning“ durchführen, wäre es zu überlegen, ob eine doch zeitlich sehr aufwendige und mit Kosten verbundene ACP-Schulung eine entscheidende Bedingung für die Durchführung der Gespräche darstellt oder ob eine Gesprächsführung mit konsequenter Anwendung des Leitfadens in Zukunft ausreichend ist.

## Fazit für die Praxis

Eine Demenz konfrontiert die Betroffenen mit gesundheitlichen und sozialen Entscheidungen. Eine Teilnahme am Entscheidungsprozess ist aufgrund der Progression der Erkrankung limitiert. Eine schriftliche Entscheidungshilfe in Verbindung mit Beratungsgesprächen als Unterstützungsangebot hilft bei der Identifikation wichtiger Vorausplanungen und begleitet bei Bedarf den Entscheidungsprozess, ohne die Betroffenen zu einer Entscheidung zu drängen. Ein niederschwelliger Zugang des Angebots in der Regelversorgung zur Stärkung der Autonomie ist anzustreben.

## Caption Electronic Supplementary Material


