# Supplementary material for: Development of a decision aid for participative advance planning for people with dementia and their relatives
Source: Nervenarzt. 2020 Apr 28;91(11):1032–9. [Article in German] doi: 10.1007/s00115-020-00911-2 (PMC7606278; doi:10.1007/s00115-020-00911-2)
Supplement: Supplementary file 1 [file 115_2020_911_MOESM1_ESM.pdf]

Klinikum rechts der Isar
Technische Universität München

LMU
LUDWIG-
MAXIMILIANS-
UNIVERSITÄT
MÜNCHEN

JETZT ENTSCHEIDUNGEN FÜR DIE ZUKUNFT TREFFEN

JETZT!

Demenz im Frühstadium – Wichtige gesundheitliche und soziale Entscheidungen rechtzeitig treffen

Eine Orientierungs- und Entscheidungshilfe für Menschen mit einer Demenz und deren Angehörige

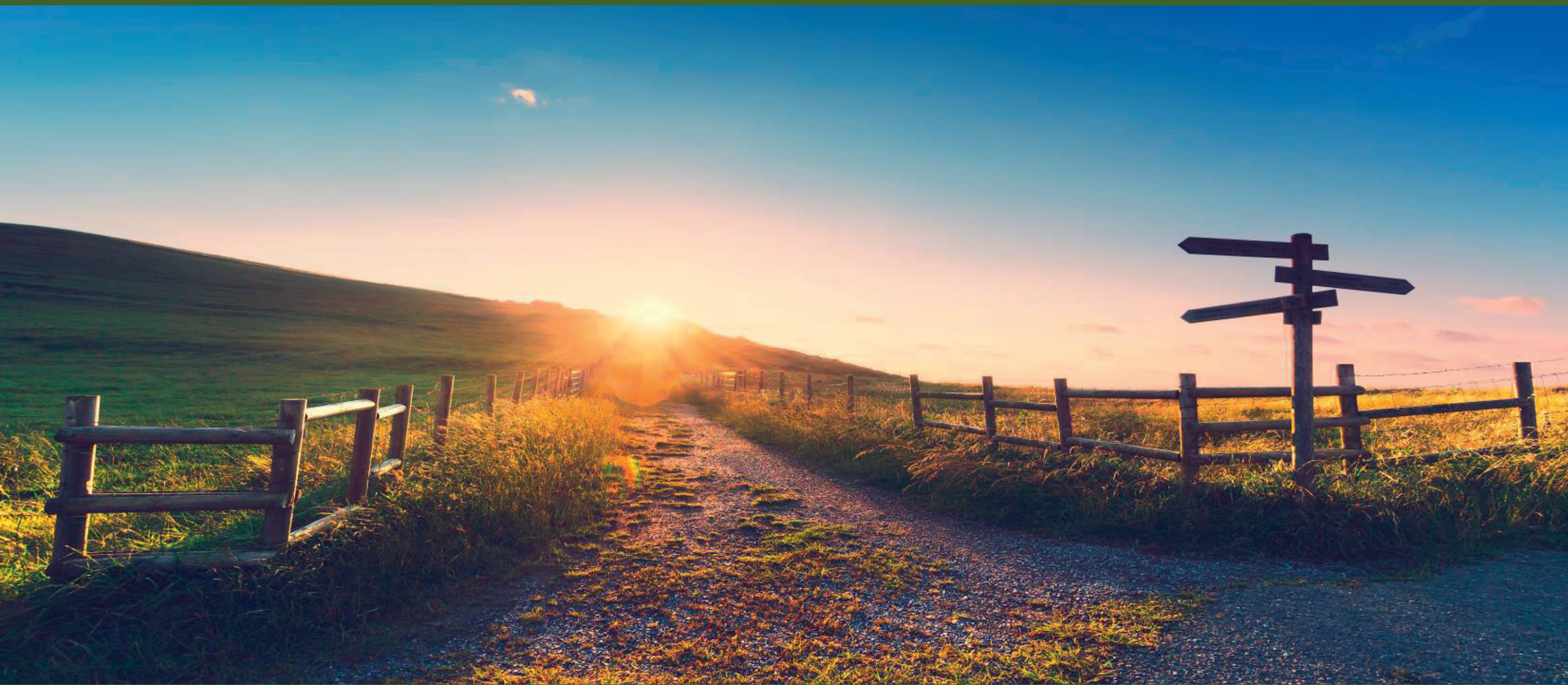

Gefördert durch DFG Deutsche Forschungsgemeinschaft

Inhaltsverzeichnis

Vorwort ... 4

Die Idee dieser Orientierungs- und Entscheidungshilfe ... 5

Übersicht über wichtige Entscheidungen der Planung und Vorsorge ... 8

Thema: Vorsorgevollmacht ... 9

- Häufig gestellte Fragen – Vorsorgevollmacht ... 10
- Entscheidungsfindung – Vorsorgevollmacht ... 15
- Vorsorgevollmacht – Was kann ich tun und wie kann ich vorgehen? ... 20

Thema: Patientenverfügung ... 23

- Häufig gestellte Fragen – Patientenverfügung ... 24
- Entscheidungsfindung – Patientenverfügung ... 29
- Patientenverfügung – Was kann ich tun und wie kann ich vorgehen? ... 34

Thema: Wohnen und Versorgung ... 37

- Häufig gestellte Fragen – Wohnen und Versorgung ... 38
- Entscheidungsfindung – Wohnen und Versorgung ... 44
- Wohnen und Versorgung – Was kann ich tun und wie kann ich vorgehen? ... 49

Thema: Autofahren und Fahreignung ... 51

- Häufig gestellte Fragen – Autofahren und Fahreignung ... 52
- Entscheidungsfindung – Autofahren und Fahreignung ... 56
- Autofahren und Fahreignung – Was kann ich tun und wie kann ich vorgehen? ... 61

Weitere Vorausplanungen und Entscheidungen ... 65

- Versicherungen ... 66
- Mitgliedschaften in Vereinen und Verbänden ... 67
- Schwerbehindertenausweis ... 67
- Beruf und Rente ... 68
- Nachlass ... 69
- Bestattung ... 69
- Forschungsverfügung für späte Stadien einer Demenz ... 70
- Organspende und Gewebespende ... 71

Überblick über Ihren aktuellen Stand der Bearbeitung der verschiedenen Themen ... 73

Vorwort

Diese Orientierungs- und Entscheidungshilfe richtet sich an **Menschen mit einer Demenz im Frühstadium und an deren Angehörige.** Sie wurde von einem Expertenteam der Technischen Universität und der Ludwig-Maximilians-Universität München verfasst. Beratend zur Seite stand eine größere Gruppe von Fachleuten aus den Bereichen Pflege, Sozialarbeit, Seelsorge, Allgemeinmedizin, Psychiatrie sowie Patienten- und Angehörigenvertretern.

Ziel dieser Orientierungs- und Entscheidungshilfe ist es, Betroffene und ihre Familien über wichtige gesundheitliche und soziale Entscheidungen zu informieren und eine persönliche Auseinandersetzung mit diesen Themen zu fördern.

Die Orientierungs- und Entscheidungshilfe:

JETZT!

INFORMIERT Menschen mit einer Demenz und deren Angehörige über gesundheitliche und soziale Entscheidungen.

STELLT Beweggründe für und gegen eine Entscheidung **ZUR VERFÜGUNG.**

ERMUTIGT Menschen mit einer Demenz und deren Angehörige, rechtzeitig Entscheidungen über wichtige gesundheitliche und soziale Fragen zu treffen.

BEGLEITET Menschen mit einer Demenz und deren Angehörige durch den Orientierungs- und Entscheidungsprozess.

Die Idee dieser Orientierungs- und Entscheidungshilfe

Im Leben kann es immer wieder Zeiten geben, in denen es darum geht, wichtige persönliche Entscheidungen für die Zukunft zu treffen und mögliche Weichen rechtzeitig zu stellen. Wir tun dies etwa bei der Berufswahl, bei Abschlüssen von Versicherungen oder im Alltag, wenn wir zum Beispiel Urlaubsreisen buchen. Auch wer eine chronische Krankheit hat, macht oft die Erfahrung, wie wichtig es ist, Behandlung, Pflege und Lebensführung rechtzeitig zu planen.

Viele Menschen mit Demenz können ihren Alltag lange weitgehend selbstbestimmt führen. Dennoch kann es im Verlauf der Demenz zunehmend schwieriger werden, vor allem komplexe Entscheidungen selbst zu treffen. Dazu gehören zum Beispiel rechtliche Angelegenheiten. Außerdem zählt dazu auch zu planen, wo man wohnt und wie man versorgt wird. Für viele dieser Entscheidungen ist es jedoch notwendig, zu diesem Zeitpunkt geschäftsfähig und einwilligungsfähig zu sein.

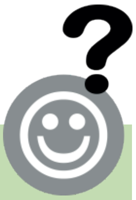

Geschäftsfähig und einwilligungsfähig sein – Was heißt das?

Man ist **geschäftsfähig**, wenn man die Bedeutung und die Tragweite von Entscheidungen, Käufen und Verträgen einschätzen kann.

Man ist **einwilligungsfähig,** wenn man in eine medizinische Behandlung einwilligen kann oder sie ablehnen kann. Dazu muss man verstehen, welche Folgen eine ärztliche Behandlung oder der Verzicht darauf haben kann. Ein Beispiel: Die Folge einer Knie-Operation kann eine Entzündung sein. Wenn man auf die Knie-Operation verzichtet, dann können Schmerzen zum Beispiel schlimmer werden. Nur wenn Sie diese Tragweite verstehen, dann können Sie einen eigenen, freien Entschluss fassen.

Um Zweifel auszuräumen und um Streit in der Familie zu vermeiden, können Sie sich Ihre Geschäftsfähigkeit und Einwilligungsfähigkeit bei einem Arzt prüfen und bestätigen lassen.

Viele Menschen mit Demenz sind bis an ihr Lebensende geschäftsfähig und einwilligungsfähig. Es gibt aber auch Betroffene, die im Laufe der Demenz nicht mehr geschäftsfähig und einwilligungsfähig sind. Da man das am Anfang der Erkrankung nicht weiß, ist es sinnvoll, wichtige Entscheidungen für die Zukunft rechtzeitig zu treffen. Allerdings fällt dies vielen Menschen schwer. Gerade wenn die Zukunft noch weit weg zu sein scheint. Deshalb schieben wir solche Entscheidungen gerne auf. Für viele Menschen kann es jedoch sehr gut sein, Entscheidungen frühzeitig anzugehen.

Es ist daher ratsam, dass Sie sich früh mit komplexen Entscheidungen für die Zukunft befassen. So haben Sie mehr Möglichkeiten Ihrer Selbstbestimmung Ausdruck zu verleihen. Außerdem geben Sie Ihren Angehörigen damit eine Hilfestellung und können ihnen eine schwere Belastung ersparen. Denn oft sind es die Angehörigen, die später einmal für Sie Entscheidungen treffen müssen, falls Sie es selbst nicht mehr können.

JETZT – Das Wichtigste in Kürze

JETZT!

- **Eigene Zukunft selbst bestimmen und planen**
- **Eigene Wünsche äußern**
- **Sich selbst und Angehörige entlasten**
- **Ungewissheit verringern**
- **Orientierung für Ärzte, Pflegende und andere Helfer bieten**

Was bietet Ihnen *JETZT*?

Diese Orientierungs- und Entscheidungshilfe unterstützt Sie und Ihre Angehörigen, sich mit der Vorausplanung von verschiedenen, wichtigen Entscheidungen für die Zukunft zu beschäftigen. Dabei gibt es keine richtigen oder falschen Entscheidungen. Entscheidungen treffen bei *JETZT* bedeutet vielmehr, dass Sie sich mit einem Thema auseinandersetzen. Sie können sich dann bewusst dafür oder dagegen entscheiden, das Thema weiter zu vertiefen. Es geht also darum, dass Sie Ihre Zukunft so gestalten können, wie es sich für Sie richtig anfühlt. So können Sie Ihre ganz persönlichen Antworten auf wichtige Fragen finden. Diese Fragen können zum Beispiel sein:

- Möchte ich eine Vorsorgevollmacht erteilen?
- Möchte ich eine Patientenverfügung erstellen?
- Möchte ich jetzt planen, wie ich später wohne und versorgt werde?
- Möchte ich jetzt prüfen, ob ich noch sicher Auto fahren kann?

JETZT fasst zu diesen Fragen die wichtigsten Informationen zusammen. Sie können sich Ihre eigenen Werte und Vorstellungen bewusst machen und dadurch soll es Ihnen leichter fallen, Entscheidungen für sich selbst zu treffen.

JETZT beschäftigt sich ausschließlich mit Themen der Vorausplanung von gesundheitlichen und sozialen Entscheidungen. Diese Themen liegen noch in der Zukunft. Sie sind nicht unbedingt sofort nach der Diagnosestellung von Bedeutung.

Unmittelbar nach der Diagnose einer Demenz stehen auch Entscheidungen zur medizinischen Behandlung an. Zu diesen Fragen finden Sie bei *JETZT* keine weiteren Informationen. Entscheidungen zu medizinischen Behandlungen, wie zum Beispiel Medikamente oder andere nicht-medikamentöse Behandlungen, können Sie zusammen mit Ihrem Arzt treffen.

Übersicht über wichtige Entscheidungen der Planung und Vorsorge

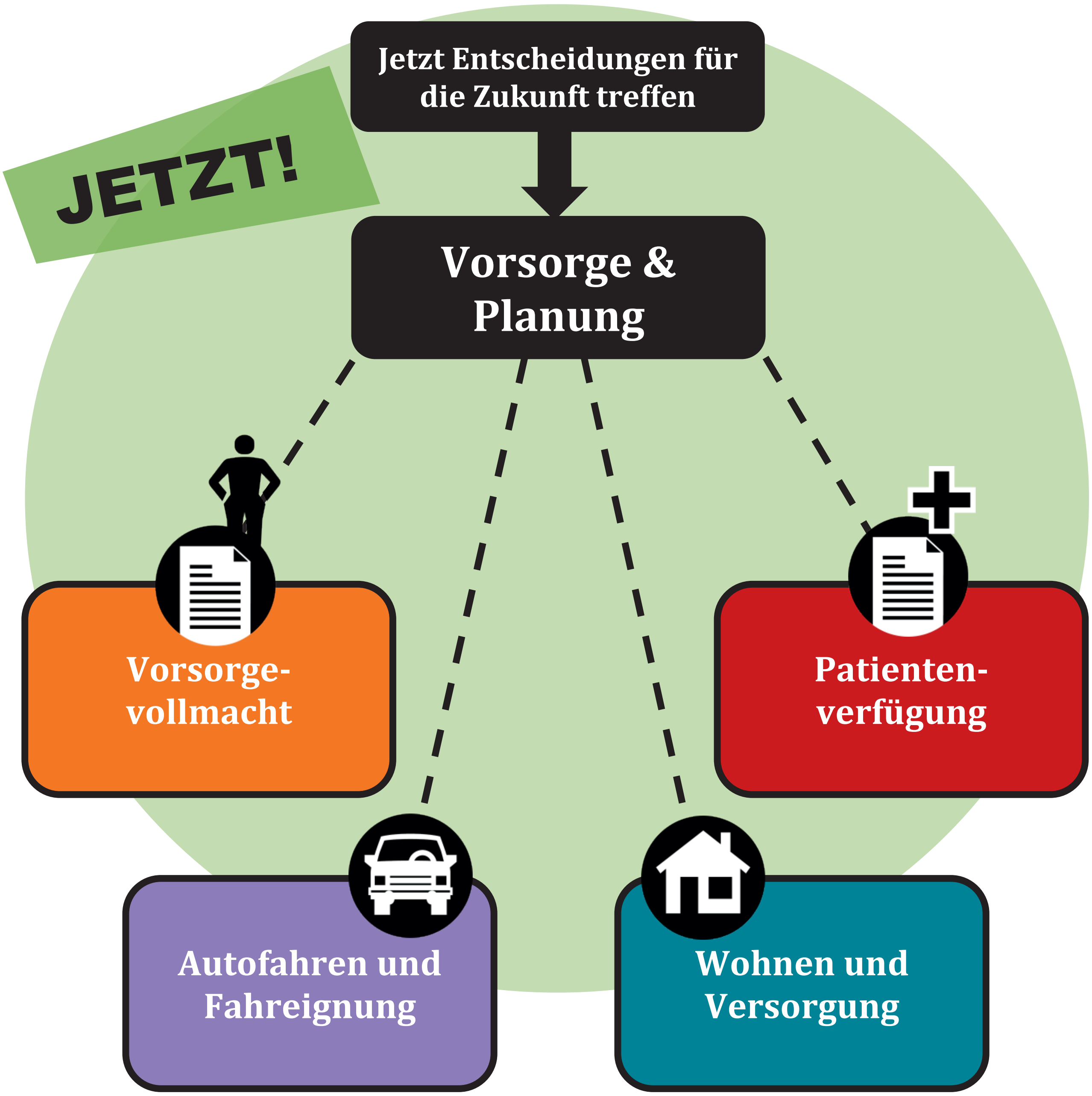

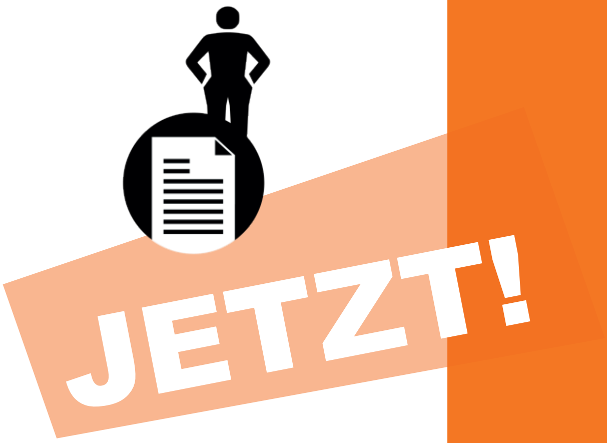

Vorsorgevollmacht

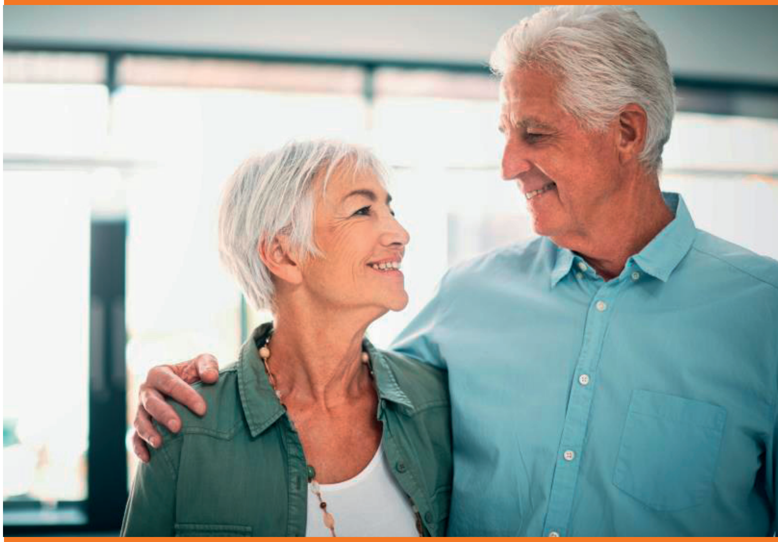

Thema: Vorsorgevollmacht

→ **Möchte ich eine Vorsorgevollmacht erteilen?**

Häufig gestellte Fragen – Vorsorgevollmacht

Was versteht man unter einer Vorsorgevollmacht?

Eine Vorsorgevollmacht ist ein schriftliches Dokument. Damit erlauben Sie einer anderen Person Entscheidungen für Sie zu treffen oder für Sie zu handeln. Diese Person wird Bevollmächtigter genannt. Sie können dabei eine oder mehrere Vertrauenspersonen benennen. Es ist wichtig zu wissen, dass Ihre Ehepartner und Verwandte auch in einem Notfall nicht automatisch für Sie Entscheidungen treffen dürfen.

Falls Sie keine Vorsorgevollmacht erteilen, dann kann folgendes passieren: Wenn Sie selbst keine Entscheidungen mehr treffen können, dann muss ein Gericht einen rechtlichen Betreuer für Sie bestellen. Dies benötigt Zeit und kostet Geld. Der Betreuer soll nach dem Gesetz eine Vertrauensperson aus der Familie sein. Wenn in Ihrer Familie keiner Ihrer Angehörigen dafür geeignet ist, wählt das Gericht eine Person für Sie aus. Das nennt man dann Berufsbetreuer. Den Berufsbetreuer kennt man vorher meist nicht.

Welche Form muss die Vorsorgevollmacht haben?

Sie können die Vorsorgevollmacht selbst schreiben oder ein bestehendes Musterformular verwenden. Das kann zum Beispiel das Musterformular der Münchener Betreuungsstelle sein. Die Adresse finden Sie am Ende des Themas. Ein Musterformular bietet den Vorteil, dass alle wesentlichen Informationen dort aufgeführt sind. Versehen Sie die Vorsorgevollmacht mit einem Datum und Ihrer Unterschrift. Eine Vorsorgevollmacht sollte keine besonderen Bedingungen enthalten, damit sie auch sofort nach der Ausstellung gültig ist. So kann der Bevollmächtigte auch in Notfällen unmittelbar für Sie entscheiden.

Bei komplizierten rechtlichen Angelegenheiten sollten Sie sich von einem Rechtsanwalt oder Notar beraten lassen. Das kann zum Beispiel der Fall sein, wenn Sie etwa ein umfangreiches Vermögen besitzen oder mehrere Bevollmächtigte einsetzen. Wenn Sie in der Vorsorgevollmacht sehr genaue Anweisungen für den Bevollmächtigten festlegen, dann sollten Sie sich auch beraten lassen.

Eine notarielle Beglaubigung oder Beurkundung ist nur für wenige Bereiche zwingend erforderlich, zum Beispiel wenn ein Haus verkauft oder Geld bei einer Bank geliehen werden soll. Man kann die Unterschrift auf der Vorsorgevollmacht zum Beispiel bei einem Notar oder bei einer Betreuungsstelle Ihrer Stadt oder Ihres Landkreises beglaubigen lassen. Dadurch bestehen dann keine Zweifel mehr an Ihrer Unterschrift oder an Ihrer Person.

Um eine gültige Vorsorgevollmacht erteilen zu können, müssen Sie geschäftsfähig sein. Weitere Informationen zum Thema Geschäftsfähigkeit finden Sie in der Einleitung. Solange Sie geschäftsfähig sind, können Sie Ihre Vorsorgevollmacht auch jederzeit ändern oder widerrufen.

Welchen Inhalt und welchen Umfang hat eine Vorsorgevollmacht?

In welchen Bereichen Sie Ihre Vollmacht erteilen wollen, können Sie frei bestimmen. Die bevollmächtigte Person kann das Recht bekommen, umfassend zu entscheiden („Generalvollmacht“) oder nur für einzelne Bereiche zuständig sein. Die wichtigsten Bereiche sind:

- Gesundheit. Zum Beispiel Arzt wählen, über Behandlung und Pflege entscheiden, Informationen von behandelnden Ärzten erhalten.
- Vermögen. Zum Beispiel Konto führen, Rechnungen bezahlen.
- Aufenthalt und Wohnen. Zum Beispiel über Umzug in eine Pflegeeinrichtung entscheiden.
- Post und Telefon. Zum Beispiel Post und E-Mails lesen, Telefonvertrag abschließen.
- Behörden und Versicherungen. Zum Beispiel Anwalt bestellen, Ausweis beantragen.

Es ist außerdem von Vorteil, wenn die Vorsorgevollmacht über den Tod hinaus erteilt wird. Nur dann kann der Bevollmächtigte bestehende Rechnungen wie zum Beispiel die Bestattungskosten von Ihrem Konto bezahlen. Sonst müssen Ihre Erben die Rechnungen so lange aus eigener Tasche bezahlen, bis ein Erbschein erteilt ist. Das kann mehrere Wochen dauern.

Sonderfall: Bankvollmacht

Manchmal bestehen Banken auf eine extra Bankvollmacht und auf ihre eigenen Formulare. Auch dann, wenn eine gültige Vorsorgevollmacht über vermögensrechtliche Angelegenheiten vorliegt. Nehmen Sie am besten Kontakt zu ihrer Bank auf und lassen Sie sich dort beraten.

Welche Personen kommen als Bevollmächtigte in Frage?

Sie sollten nur Personen bevollmächtigen, zu denen Sie ein großes Vertrauen haben. Das können zum Beispiel Familienangehörige oder enge Freunde sein. Denn der Bevollmächtigte wird bei einer Vorsorgevollmacht eigenverantwortlich tätig und nicht automatisch durch das Gericht überwacht. Da die übertragenen Aufgaben für einen Bevollmächtigten manchmal sehr belastend sein können, sollten Sie die Person Ihres Vertrauens vorher fragen, ob derjenige diese Aufgabe übernehmen möchte. Auch sollten Sie diese Person umfassend über Ihre Wünsche informieren. Nur so kann diese Person Ihre Wünsche kennen und danach handeln. Eine Vorsorgevollmacht kann auch abgelehnt werden.

Ab wann ist eine Vorsorgevollmacht gültig?

Eine Vorsorgevollmacht ist sofort mit der Erteilung wirksam. Voraussetzung dafür ist, dass die Vorsorgevollmacht im Original vorgelegt wird. Solange Sie das Original der Vorsorgevollmacht in Ihrem Besitz haben, kann der Bevollmächtigte nicht handeln. Eine Kopie genügt nicht.

Was versteht man unter einer Betreuungsverfügung?

Neben der Vorsorgevollmacht gibt es noch eine andere Möglichkeit für den Fall vorzusorgen, wenn Sie selber nicht mehr in der Lage sind Entscheidungen zu treffen. Diese Möglichkeit nennt sich „Betreuungsverfügung“. In einer Betreuungsverfügung können Sie eine Person vorschlagen, die Sie sich als rechtlichen Betreuer wünschen. Sie können auch Personen benennen, die Sie als rechtliche Betreuer ablehnen.

Bei einer Betreuungsverfügung handelt es sich um einen Vorschlag für einen rechtlichen Betreuer an das Gericht. Das Gericht ist grundsätzlich an diesen Vorschlag gebunden. Es kann sein, dass die Person, die Sie in der Betreuungsverfügung als rechtlichen Betreuer vorgeschlagen haben, manchmal vom Gericht nicht eingesetzt wird. Dazu muss das Gericht jedoch gute Gründe haben.

Es gibt folgende Unterschiede zur Vorsorgevollmacht:

- Der rechtliche Betreuer kann erst für Sie handeln, wenn er durch das Gericht bestellt ist.
- Der rechtliche Betreuer untersteht einer regelmäßigen Kontrolle des Gerichts.
- Das Gericht bestimmt, welche Bereiche die Betreuung umfasst.
- Es fallen für Sie regelmäßige Kosten für den rechtlichen Betreuer sowie Gerichtskosten an.

Vorsorgevollmacht – Das Wichtigste in Kürze

- Sie bestimmen selber, wer für Sie später einmal Entscheidungen treffen darf.
- Eine Vorsorgevollmacht ist erst dann wirksam, wenn Ihre Vertrauensperson (Bevollmächtigter) die Vorsorgevollmacht im Original vorlegt.
- Sie können eine umfassende Vorsorgevollmacht („Generalvollmacht“) erteilen oder nur einzelne Bereiche benennen.
- Wenn Sie eine Vorsorgevollmacht erteilen, dann müssen Sie zum Zeitpunkt der Erteilung geschäftsfähig sein.
- Wenn Sie nicht mehr für sich entscheiden können und keine Vorsorgevollmacht haben, dann wird das Gericht einen rechtlichen Betreuer für Sie einsetzen.
- Sie sollten den Bevollmächtigten über die Vollmacht und Ihre Wünsche informieren, damit er diese kennt und umsetzen kann.
- Der Bevollmächtige kann diese Aufgabe auch jederzeit ablehnen.
- Alternativ zur Erteilung der Vorsorgevollmacht gibt es auch noch die Möglichkeit, eine Betreuungsverfügung zu verfassen.

Entscheidungsfindung – Vorsorgevollmacht

Im Folgenden sehen Sie eine Tabelle. Auf der linken Seite in **grün** lesen Sie, was Menschen bewegt, eine Vorsorgevollmacht zu erteilen. Auf der rechten Seite in **blau** lesen Sie, was Menschen bewegt, keine Vorsorgevollmacht zu erteilen.

**Was bewegt Menschen, eine Vorsorgevollmacht zu erteilen?**	**Was bewegt Menschen, keine Vorsorgevollmacht zu erteilen?**
Es soll jemand, insbesondere auch in einem Notfall, schnell für mich entscheiden können.<br><br>Ich kann die Person selbst bestimmen, die für mich Entscheidungen trifft, wenn es notwendig ist.<br><br>Ich kann mit meinem Bevollmächtigten sprechen und ihm meine Werte und Wünsche mitteilen.<br><br>Ich kann selbst die Bereiche der Vorsorgevollmacht bestimmen.<br><br>Ich spare Zeit, Aufwand und Kosten, die mit einer rechtlichen Betreuung durch das Gericht verbunden wären.	Ich kenne keine Person, der ich so umfassend vertraue.<br><br>Ich möchte diese Verantwortung und die eventuell schwierigen Entscheidungen keiner Person zumuten.<br><br>Ich möchte, dass erst durch das Betreuungsgericht geprüft wird, ob ich eine rechtliche Betreuung benötige und in welchem Umfang sie erforderlich ist.<br><br>Ich möchte eine Kontrolle meines Vertreters durch das Gericht.

Persönliche Ansichten von Menschen mit Demenz und deren Angehörige:

Im Folgenden lesen Sie Aussagen von Menschen, die sich mit dem Thema Vorsorgevollmacht auseinandergesetzt haben. Es handelt sich dabei um ganz persönliche Ansichten der jeweiligen Personen.

„Es war mir wichtig, dass ich meinen Vorsorgebevoll-mächtigten selber bestimmen konnte.“

Betroffener

„In meinem Umfeld gibt es niemanden, dem ich richtig vertrauen kann.“

Betroffene

„Ich konnte sofort im Namen meiner Mutter handeln und offene Rechnungen bezahlen, als sie dazu nicht mehr in der Lage war. Das war unkompli-ziert und hat uns die Situation sehr erleichtert.“

Angehörige

„Ich mache mir Sorgen, dass ich meine Tochter mit dieser Aufgabe überfordern könnte, weil sie selber so beschäftigt ist.“

Angehöriger

Ihr Gefühl – Vorsorgevollmacht

Was ist für Sie besonders wichtig? Überlegen Sie, was für Sie mehr zutrifft und kreuzen Sie es dementsprechend an.

Ich möchte die Vertrauensperson und die Bereiche der Vorsorgevollmacht selber bestimmen.

voll und ganz	eher ja	teils/teils	eher nein	überhaupt nicht

Ich habe eine oder mehrere Vertrauenspersonen, denen ich zutraue, dass sie meine Wünsche berücksichtigen und zu meinem Wohle entscheiden werden.

voll und ganz	eher ja	teils/teils	eher nein	überhaupt nicht

Ich überfordere meine Vertrauensperson mit dieser Aufgabe nicht.

voll und ganz	eher ja	teils/teils	eher nein	überhaupt nicht

Ihre Einstellung – Vorsorgevollmacht

Wenn Sie sich mit dem Thema Vorsorgevollmacht beschäftigt haben, dann haben Sie sich vielleicht schon eine Meinung gebildet. Bitte kreuzen Sie Ihre aktuelle Einstellung an.

Möchte ich eine Vorsorgevollmacht erteilen?

voll und ganz	eher ja	teils/teils	eher nein	überhaupt nicht

Ihre Notizen – Vorsorgevollmacht

Schreiben Sie bitte in das untenstehende Feld Ihre Fragen, Anmerkungen, Bedenken und nächsten Schritte zum Thema Vorsorgevollmacht. Sie können dieses Textfeld auch freilassen.

Vorsorgevollmacht – Frage an den Angehörigen

Wenn Sie sich mit dem Thema Vorsorgevollmacht beschäftigt haben, dann haben Sie sich vielleicht schon eine Meinung gebildet. Bitte kreuzen Sie Ihre aktuelle Einstellung an.

Soll Ihr Angehöriger eine Vorsorgevollmacht erteilen?

voll und ganz	eher ja	teils/teils	eher nein	überhaupt nicht

Ihre Notizen – Vorsorgevollmacht

Schreiben Sie bitte in das untenstehende Feld Ihre Fragen, Anmerkungen, Bedenken und nächsten Schritte zum Thema Vorsorgevollmacht. Sie können dieses Textfeld auch freilassen.

Betreuungsverfügung

Der Abschnitt „Vorsorgevollmacht – Was kann ich tun und wie kann ich vorgehen“ bezieht sich genauso auf die Betreuungsverfügung.

Wo kann ich mich zum Thema Vorsorgevollmacht beraten lassen?

Beratung zu Vorsorgevollmacht bieten zum Beispiel viele Sozialvereine an. Sie können sich auch an die Betreuungsstellen wenden. Diese sind im Landratsamt oder im Kreisverwaltungsreferat der großen Städte zu finden. Bei komplexen Fragestellungen, mehreren Bevollmächtigten oder größeren Vermögenswerten empfehlen wir eine fachliche Beratung bei einem Anwalt oder Notar.

Gibt es Musterformulare für eine Vorsorgevollmacht?

Es gibt zahlreiche Musterformulare für die Vorsorgevollmacht oder Betreuungsverfügung. Sie finden diese zum Beispiel auf den Internet-Seiten der Betreuungsstelle der Landeshauptstadt München, des Bayrischen Justizministeriums und des Bundesministeriums der Justiz und für Verbraucherschutz. Außerdem finden Sie dort Informationen zur Bankvollmacht.

Wo bewahre ich meine Vorsorgevollmacht auf?

Handeln kann die benannte Person von Ihrer Vorsorgevollmacht nur, wenn diese Person die Vorsorgevollmacht im Original oder eine Ausfertigung der notariellen Urkunde vorweisen kann. Informieren Sie diese Person, wo Sie Ihre Vorsorgevollmacht oder Betreuungsverfügung und auch weitere wichtige Unterlagen aufbewahren.

Bewahren Sie außerdem in Ihrem Geldbeutel eine Informationskarte auf, auf der steht, dass Sie eine Vorsorgevollmacht haben und wo dieses Dokument zu finden ist. Zugleich sollte dort die Telefonnummer der Person zu finden sein, die im Notfall sofort zu benachrichtigen ist und Ihre Vorsorgeverfügungen kennt.

Kann ich meine Vorsorgevollmacht registrieren lassen?

Sie können Ihr Dokument gegen eine einmalige Gebühr von etwa 20 Euro beim Zentralen Vorsorgeregister der Bundesnotarkammer lebenslang registrieren lassen. Dort wird die Information hinterlegt, dass Sie eine Vorsorgevollmacht oder Betreuungsverfügung erteilt haben. Dadurch kann vermieden werden, dass ein Richter einen rechtlichen Betreuer einsetzt, nur weil die Vorsorgevollmacht nicht bekannt war.

Vorsorgevollmacht – Wichtige Anlaufstellen und Adressen

Landeshauptstadt München Sozialreferat

https://www.muenchen.de/rathaus/Stadtverwaltung/Sozialreferat/Sozialamt/Betreuungsstelle/dokumente.html

→ Hier erhalten Sie weitere Informationen zur Vorsorgevollmacht und Betreuungsverfügung, Musterformulare und eine Liste der Münchner Betreuungsvereine.

Bayerisches Staatsministerium der Justiz und für Verbraucherschutz

https://www.justiz.bayern.de/

→ Hier finden Sie Informationen zur Vorsorgevollmacht und Betreuungsverfügung und eine Broschüre zum Betreuungsrecht, sowie Musterformulare.

Bundesministeriums der Justiz und für Verbraucherschutz:

www.bmjv.de

→ Hier finden Sie eine Broschüre zum Thema Betreuungsrecht und Musterformulare zu Vorsorgevollmacht, Betreuungsverfügung und Konto- und Depotvollmacht.

Bundesnotarkammer:

www.vorsorgeregister.de

→ Hier können Sie Ihre Vorsorgevollmacht oder Betreuungsverfügung registrieren lassen.

JETZT!

Patientenverfügung

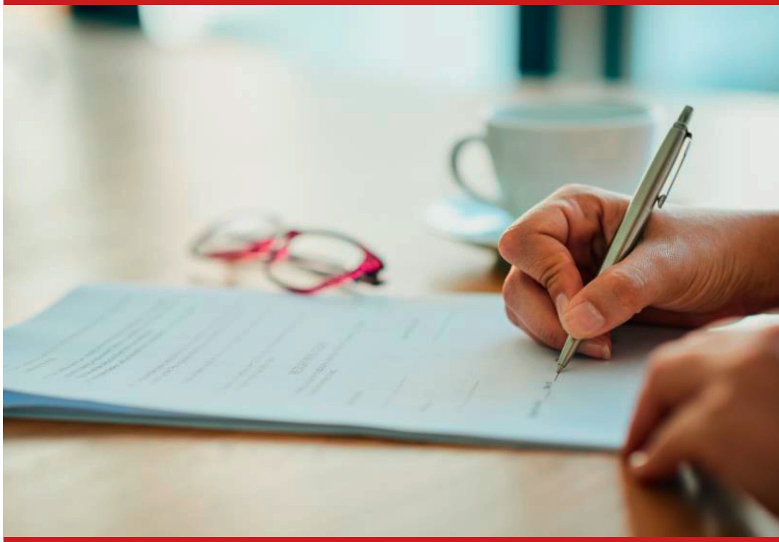

Thema: Patientenverfügung

→ **Möchte ich eine Patientenverfügung erstellen?**

Häufig gestellte Fragen – Patientenverfügung

Was versteht man unter einer Patientenverfügung?

Eine Patientenverfügung ist eine Anweisung an Ärzte und andere Personen, ob und wie Sie zukünftig behandelt werden wollen. Sie kommt nur dann zum Einsatz, wenn Sie selbst nicht mehr sagen oder entscheiden können, ob Sie in eine bestimmte Behandlung einwilligen oder nicht. Das kann zum Beispiel der Fall sein, wenn Ihre Demenz weit fortgeschritten ist oder Sie einen Unfall hatten oder für längere Zeit bewusstlos sind.

In einer Patientenverfügung können Sie festlegen, welche Behandlungen bei Ihnen vorgenommen werden dürfen und welche nicht. Das kann zum Beispiel eine künstliche Ernährung oder die Gabe bestimmter Medikamente sein, wie zum Beispiel ein Antibiotikum gegen eine schwere Infektion. Häufig geht es dabei um medizinische Maßnahmen, die das Leben verlängern sollen.

Es ist sinnvoll, dass Sie Ihre Patientenverfügung mit einer Vorsorgevollmacht oder Betreuungsverfügung ergänzen. So kann Ihre Vertrauensperson oder Ihr rechtlicher Betreuer Ihre Patientenverfügung und somit Ihren Willen gegenüber den Ärzten durchsetzen. Besprechen Sie Ihre Wünsche und Vorstellungen mit Ihrer Familie oder Ihrer Vertrauensperson, damit die für Sie richtigen Entscheidungen getroffen werden können. Gesprochene Worte sind eine sinnvolle Ergänzung zum Text der Patientenverfügung.

Es ist sehr wichtig, dass Sie Ihre Patientenverfügung regelmäßig überprüfen. Sie können die Patientenverfügung jederzeit wieder für ungültig erklären und neu verfassen.

Welche Form sollte eine Patientenverfügung haben?

Sie müssen eine Patientenverfügung schriftlich erstellen und eigenhändig mit Namen und Datum unterschreiben. Sie können die Patientenverfügung zusätzlich von einem Zeugen unterschreiben lassen. Dieser kann dann bestätigen, dass Sie zum Zeitpunkt der Erstellung einwilligungsfähig waren. So können Sie verhindern, dass Ihre Festlegungen angezweifelt werden.

Es ist wichtig, dass Sie die Festlegungen in Ihrer Patientenverfügung möglichst genau treffen. Sie müssen konkrete gesundheitliche Situationen beschreiben oder konkrete Behandlungsmaßnahmen benennen, die Sie ablehnen beziehungsweise wünschen. Am besten benennen Sie beides, Situationen und Maßnahmen, genau.

Ist eine Beratung zum Thema Patientenverfügung sinnvoll?

Häufig nutzen Menschen eine Patientenverfügung, um medizinische Maßnahmen in Situationen einer sehr schweren Krankheit oder sogar am Lebensende abzulehnen. Es geht dabei zum Beispiel um die Frage der künstlichen Beatmung bei einer schweren Lungenerkrankung. Ihre Entscheidung kann weitreichende Folgen haben. Deswegen sollten Sie die Patientenverfügung mit Ihrem Hausarzt besprechen.

Muss eine Patientenverfügung vom Arzt befolgt werden?

Jeder Arzt ist gesetzlich an die Festlegungen in Ihrer Patientenverfügung gebunden, wenn diese Festlegungen auf Ihre dann aktuelle Situation zutreffen

Deswegen muss stets genau geprüft werden, ob die Patientenverfügung auf die aktuelle Situation passt. Der Arzt prüft in einem Gespräch mit Ihrer bevollmächtigen Vertrauensperson oder Ihrem rechtlichen Betreuer, ob die Situation eingetreten ist, die Sie in der Patientenverfügung benannt haben. Oder es muss ausgelegt werden, ob nach dem Sinn der Verfügung der aktuelle Wille ermittelt werden kann.

Liegt eine gültige, eindeutig zutreffende Patientenverfügung vor, muss der Arzt sie befolgen, auch wenn kein Bevollmächtigter oder rechtlicher Betreuer vorhanden ist.

Welche künftigen Situationen sollte ich bedenken?

Sie sollten vor allem solche Situationen bedenken, die Ihre Demenz im weiteren Verlauf mit sich bringen kann. Das sind insbesondere die Fragen: Gibt es ein Stadium der Demenz oder einen gesundheitlichen Zustand, in dem Sie nicht mehr wollen, dass Ihr Leben medizinisch verlängert wird? Eine solche Lebensverlängerung kann zum Beispiel durch eine Wiederbelebung, eine Behandlung auf der Intensivstation, eine künstliche Beatmung, eine künstliche Ernährung oder die Behandlung einer Infektion mit Medikamenten geschehen.

Daneben können Sie festlegen, ob Sie eine lebensverlängernde Behandlung auch dann ablehnen, wenn andere Personen bei Ihnen Lebensfreude oder Lebenswille erkennen.

Welche Folgen kommen auf mich und meine Angehörigen zu, wenn ich keine Patientenverfügung gemacht habe?

Je weniger Informationen Ihre Angehörigen und Ärzte über Ihre Wünsche haben, umso schwerer können sie entscheiden, welche Behandlungen in Ihrem Sinne vorgenommen werden sollen oder nicht. Ohne Patientenverfügung oder eindeutige mündliche Aussagen müssen diese Personen Ihren „mutmaßlichen Willen" herausfinden. Dabei werden Ihre persönlichen Einstellungen zum Leben und sonstige frühere Äußerungen berücksichtigt. Das ist jedoch häufig schwierig, zeitraubend, belastend und kann zu Streitigkeiten führen. In einer Notfallsituation wird die Zeit dafür meist fehlen. Der Arzt muss dann so handeln, wie er es medizinisch für erforderlich hält. Im Zweifel gilt es zunächst Leben zu retten.

Sonderfall Notfallplan

In einer Notfallsituation muss oft innerhalb von Sekunden oder Minuten gehandelt werden, wie zum Beispiel bei einem Herzstillstand. Es fehlt dann in der Regel die Zeit, eine Patientenverfügung zu lesen und zu beurteilen. Deshalb gibt es den sogenannten Notfallplan: er enthält eindeutige, sofort umsetzbare Anordnungen für einen Notarzt oder Rettungssanitäter. Der Notfallplan wird deshalb von Ihrem jetzigen Arzt unterschrieben, nachdem dieser mit Ihnen darüber gesprochen hat, was Sie in solchen Notfällen wollen oder nicht.

Folgende lebensrettende Notfallmaßnahmen werden im Notfallplan angesprochen:

- Herz-Lungen-Wiederbelebung
- künstliche Beatmung
- Behandlung auf einer Intensivstation
- Einweisung in ein Krankenhaus

Es ist sehr wichtig, dass Sie sich gut informieren und sich mit Ihrem Arzt beraten, damit Ihre Festlegungen im Notfallplan auch mit Ihren Behandlungswünschen übereinstimmen.
Schließen Sie nur solche Behandlungen aus, die Sie ganz sicher nicht wollen. Ansprechpartner ist in der Regel Ihr Hausarzt.

Patientenverfügung – Das Wichtigste in Kürze

- Eine Patientenverfügung ist eine Anweisung für behandelnde Ärzte und andere Beteiligte, welche Behandlungen bei Ihnen durchgeführt werden sollen oder nicht.

- Die Patientenverfügung kommt nur dann zur Anwendung, wenn Sie selbst nicht mehr einwilligen oder ablehnen können.

- Die Festlegungen in Ihrer Patientenverfügung sind rechtlich verbindlich, wenn sie auf Ihre aktuelle Lebens- und Behandlungssituation zutreffen.

- Sie können festlegen, ab welchem gesundheitlichen Zustand bestimmte lebenserhaltende Maßnahmen nicht mehr durchgeführt werden sollen.

- Es ist wichtig, dass Sie Ihre Patientenverfügung regelmäßig überprüfen und anpassen.

- Um eine gültige Patientenverfügung verfassen zu können, müssen Sie noch einwilligungsfähig sein.

- Ohne Patientenverfügung müssen Ihre Angehörigen oder Ihr rechtlicher Vertreter Ihren Willen herausfinden und danach handeln. Dies ist häufig schwierig, zeitraubend und belastend.

Entscheidungsfindung – Patientenverfügung

Im Folgenden sehen Sie eine Tabelle. Auf der linken Seite in **grün** lesen Sie, was Menschen bewegt, eine Patientenverfügung zu erstellen. Auf der rechten Seite in **blau** lesen Sie, was Menschen bewegt, keine Patientenverfügung zu erstellen.

**Was bewegt Menschen, <u>eine Patientenverfügung</u> zu erstellen?**	**Was bewegt Menschen, <u>keine Patientenverfügung</u> zu erstellen?**
Ich bestimme selbst, ob ärztliche Behandlungen bei mir durchgeführt werden sollen oder nicht.<br><br>Ich bin bereit, mich mit den Themen „Hilflosigkeit, Sterben und Tod" intensiv auseinanderzusetzen.<br><br>Ich möchte diejenigen, die die Entscheidungen treffen (zum Beispiel meine Angehörigen) entlasten, indem ich meine Wünsche auch schriftlich mitteile.	Es belastet und überfordert mich zu sehr, mich mit meinen eigenen persönlichen Einstellungen, Ängsten und Hoffnungen zu Themen wie Krankheit, Leiden und Tod zu beschäftigen.<br><br>Ich kenne mich noch zu wenig mit dem Thema aus und benötige noch weitere Informationen.<br><br>Ich vertraue darauf, dass meine rechtlichen Vertreter für mich gute Entscheidungen treffen werden, auch wenn ich keine Patientenverfügung erstellt habe.

Persönliche Ansichten von Menschen mit Demenz und deren Angehörigen

Im Folgenden lesen Sie Aussagen von Menschen, die sich mit dem Thema Patientenverfügung auseinandergesetzt haben. Es handelt sich dabei um ganz persönliche Ansichten der jeweiligen Personen.

„Es wäre für mich unerträglich, wenn man mich nicht sterben ließe, wenn meine Demenz so weit fortgeschritten ist, dass ich meine Liebsten nicht mehr erkenne.“

Betroffener

„Ich möchte mich im Moment nicht mit Dingen wie Krankheit und Tod beschäftigen.“

Betroffene

„Es war für mich leichter, diese schwierigen Entscheidungen zu treffen, weil mein Mann bereits alles in der Patientenverfügung festgelegt hatte. Das hat mich sehr entlastet.“

Angehöriger

„Ich glaube, dass die Ärzte jemanden mit Patientenverfügung eher aufgeben und ihn schlechter versorgen.“

Angehöriger

Ihr Gefühl – Patientenverfügung

Was ist für Sie besonders wichtig? Überlegen Sie, was für Sie mehr zutrifft und kreuzen Sie es dementsprechend an.

Ich will selbst festlegen, ob und wie ärztliche Maßnahmen durchgeführt werden sollen oder nicht.

voll und ganz	eher ja	teils/teils	eher nein	überhaupt nicht

Ich traue es mir zu, mich mit meinen eigenen Vorstellungen zu den Themen Leben, Krankheit und Tod auseinanderzusetzen und mir dabei helfen zu lassen.

voll und ganz	eher ja	teils/teils	eher nein	überhaupt nicht

Ich entlaste meine Angehörigen, wenn ich eine Patientenverfügung erstelle.

voll und ganz	eher ja	teils/teils	eher nein	überhaupt nicht

Eine Patientenverfügung wird zu meinem Besten angewendet und wird mir keinen Schaden bringen.

voll und ganz	eher ja	teils/teils	eher nein	überhaupt nicht

Ihre Einstellung – Patientenverfügung

Wenn Sie sich mit dem Thema Patientenverfügung beschäftigt haben, haben Sie sich vielleicht schon eine Meinung gebildet. Bitte kreuzen Sie Ihre aktuelle Einstellung an.

Möchte ich eine Patientenverfügung erstellen?

voll und ganz	eher ja	teils/teils	eher nein	überhaupt nicht

Ihre Notizen – Patientenverfügung

Schreiben Sie bitte in das untenstehende Feld Ihre Fragen, Anmerkungen, Bedenken und nächsten Schritte zum Thema Patientenverfügung. Sie können dieses Textfeld auch freilassen.

Patientenverfügung – Frage an den Angehörigen

Wenn Sie sich mit dem Thema Patientenverfügung beschäftigt haben, haben Sie sich vielleicht schon eine Meinung gebildet. Bitte kreuzen Sie Ihre aktuelle Einstellung an.

Soll Ihr Angehöriger eine Patientenverfügung erstellen?

voll und ganz	eher ja	teils/teils	eher nein	überhaupt nicht

Ihre Notizen – Patientenverfügung

Schreiben Sie bitte in das untenstehende Feld Ihre Fragen, Anmerkungen, Bedenken und nächsten Schritte zum Thema Patientenverfügung. Sie können dieses Textfeld auch freilassen.

Patientenverfügung – Was kann ich tun und wie kann ich vorgehen?

Wie erstelle ich eine Patientenverfügung?

Sie können eine Patientenverfügung frei formulieren oder auf vorhandene Formulare zurückgreifen. Es werden zahlreiche Formulare, Beispiele und Textbausteine für Patientenverfügungen angeboten. Musterformulare und Beispiele können Sie sehr gut als Anregung für eine persönliche Patientenverfügung verwenden. In vielen Fällen geben Musterformulare oder Textbausteine Ihre individuelle Situation und Ihre Wünsche jedoch nicht genau genug wieder. Wenn Sie Ankreuzformulare verwenden, sollten Sie deshalb persönliche Aussagen zu Ihrer Situation, Ihren Werthaltungen und Ihren Entscheidungen handschriftlich ergänzen.

Versehen Sie die Patientenverfügung mit Ihrem Namen, einem Datum und Ihrer Unterschrift. Eine Patientenverfügung bei einem Anwalt oder Notar zu erstellen ist möglich, aber meist nicht notwendig.

Wo kann ich mich zum Thema Patientenverfügung beraten lassen?

Sie können das Thema Patientenverfügung zum Beispiel mit Ihrem Hausarzt besprechen. Beratung zum Thema Patientenverfügung bieten auch Sozialvereine, Hospizvereine und Betreuungsstellen an.

Kann ich meine Patientenverfügung registrieren lassen?

Sie haben die Möglichkeit Ihre Patientenverfügung beim Zentralen Vorsorgeregister lebenslang gegen eine einmalige Gebühr von etwa 20 Euro registrieren zu lassen. Dazu müssen Sie aber auch eine Vorsorgevollmacht erstellt und zur Registrierung angemeldet haben. Dies wird registriert mit dem Hinweis, dass eine Patientenverfügung existiert.

Wie bekommt der behandelnde Arzt meine Patientenverfügung?

Verwahren Sie das Original Ihrer Patientenverfügung am besten so auf, dass Ihre Angehörigen, Ihr Bevollmächtigter oder Ihr rechtlicher Betreuer diese möglichst schnell finden und weitergeben können. Machen Sie zudem Kopien und geben Sie diese Ihren Angehörigen oder Ihrem behandelnden Arzt, gegebenenfalls Ihrem Pflegedienst oder anderen Menschen, die in Ihre Betreuung und Pflege eingebunden sind. Bewahren Sie am besten zusätzlich in Ihrem Geldbeutel eine Informationskarte auf, auf der steht, dass Sie eine Patientenverfügung haben und wo diese zu finden ist.

Patientenverfügung – Wichtige Anlaufstellen und Adressen

Landeshauptstadt München Sozialreferat

https://www.muenchen.de/rathaus/Stadtverwaltung/Sozialreferat/Sozialamt/Betreuungsstelle/dokumente.html

→ Hier erhalten Sie weitere Informationen und eine Broschüre zum Thema Patientenverfügung.

Bayerisches Staatsministerium der Justiz und für Verbraucherschutz

https://www.justiz.bayern.de/

→ Hier finden Sie eine Broschüre zum Thema Patientenverfügung und ein Formular für die Patientenverfügung, das sich in der Praxis bewährt hat.

Bundesministeriums der Justiz und für Verbraucherschutz:

www.bmjv.de

→ Hier finden Sie eine Broschüre zum Thema Patientenverfügung und Textbausteine für eine schriftliche Patientenverfügung.

Bundesnotarkammer:

www.vorsorgeregister.de

→ Hier können Sie Ihre Patientenverfügung registrieren lassen.

Sozialverband VdK:

www.vdk.de

→ Hier finden Sie Informationen zum Thema Patientenverfügung.

Wohnen und Versorgung

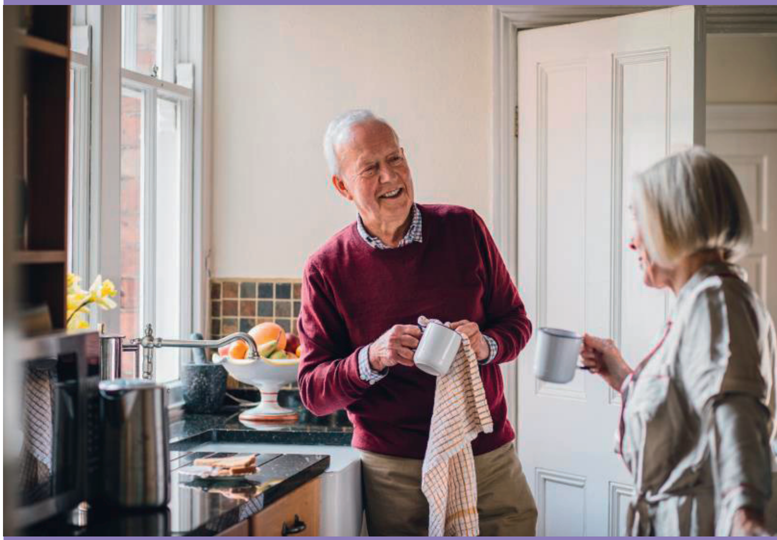

Thema: Wohnen und Versorgung

→ **Möchte ich jetzt planen, wie ich später wohne und versorgt werde?**

Häufig gestellte Fragen – Wohnen und Versorgung

Warum ist es sinnvoll, sich schon jetzt um das Thema „Wohnen und Versorgung“ zu kümmern?

Für viele Menschen mit Demenz ist es wichtig in einer vertrauten Umgebung zu wohnen, in der man sich wohl fühlt und gut aufgehoben fühlt. Sie entscheiden selbst, wie und wo Sie später wohnen und versorgt werden möchten. Wenn Sie sich mit dem Thema Wohnen und Versorgung beschäftigen, dann können Ihre Angehörigen Ihre Bedürfnisse besser verstehen und werden dadurch entlastet. Es ist oft hilfreich, sich mit den verschiedenen Möglichkeiten, wie man wohnen und versorgt werden kann, früh vertraut zu machen.

Muss man mit einer Demenz früher oder später in eine Pflegeeinrichtung ziehen?

Nein, das muss man nicht. Für viele Menschen mit Demenz ist es unvorstellbar und beängstigend, irgendwann einmal ihr vertrautes Zuhause verlassen zu müssen. Dabei können viele Menschen mit einer Demenz bis an ihr Lebensende in ihrem eigenen Zuhause wohnen bleiben. So gibt es viele Angebote der Unterstützung, um das Leben mit Demenz in den eigenen vier Wänden zu erleichtern. Das kann zum Beispiel die Pflege durch einen Angehörigen sein. Es gibt auch die Möglichkeit, dass ein Pflegedienst zu Ihnen nach Hause kommt. Manchmal kann es jedoch mit fortschreitender Demenz zu schwierig werden, eine ausreichende Versorgung zu Hause zu gewährleisten. Denn auch Angehörige und Pflegedienste können an ihre Grenzen stoßen. In solchen Fällen kann ein Umzug in eine Pflegeeinrichtung oder in ein „betreutes Wohnen“ sinnvoll sein. Es kommt immer auf die persönliche Situation jedes einzelnen an. Für den einen kann das eigene Zuhause, für den anderen der Umzug in eine Pflegeeinrichtung besser geeignet sein.

Wie kann ich meine Wohn- und Versorgungssituation zu Hause verbessern?

Es gibt verschiedene Möglichkeiten, das Leben mit Demenz in den eigenen vier Wänden zu ermöglichen.

Pflege durch einen Angehörigen

Viele Menschen mit Demenz kommen bis an ihr Lebensende ohne zusätzliche professionelle Hilfe zu Hause zurecht. Viele Menschen mit Demenz haben Angehörige, auf die sie sich verlassen und die die Versorgung übernehmen können. Angehörige, die sich um einen Menschen mit Demenz kümmern, können zusätzlich Unterstützung von Pflegediensten erhalten. Es ist jedoch wichtig zu wissen, dass die Pflege eines Menschen mit Demenz für Angehörige eine große Herausforderung darstellen und sehr belastend sein kann.

Ambulanter Pflegedienst

Menschen mit Demenz haben in der Regel Anspruch auf einen ambulanten Pflegedienst. Dabei kommt eine Pflegekraft mehrmals am Tag in die Wohnung und unterstützt zum Beispiel bei der Körperpflege oder bei der Einnahme von Medikamenten. Dafür benötigt es einen ambulanten Dienst vor Ort.

Hauswirtschaftliche Hilfen

Einige soziale Einrichtungen, Pflegedienste oder private Anbieter vermitteln Unterstützung bei gelegentlichen hauswirtschaftlichen Tätigkeiten wie etwa Wäsche waschen. Damit soll das Leben zu Hause erleichtert werden.

Besuchsdienste von ehrenamtlichen Helfern

Es kann sein, dass ehrenamtliche Helfer stundenweise die Betreuung und Begleitung von Menschen mit einer Demenz übernehmen. Dazu gehören zum Beispiel Spaziergänge, Kochen, Gesellschaftsspiele und andere gemeinsame Unternehmungen. Hauswirtschaftliche oder pflegerische Tätigkeiten werden nicht von ehrenamtlichen Helfern übernommen.

24-Stunden Pflegedienst

In fortgeschrittenen Stadien der Demenz kann eine Betreuung rund um die Uhr notwendig sein. Es gibt Pflegedienste, welche sich auf eine 24-Stunden Pflege spezialisiert haben. Dabei ist eine besonders ausgebildete und geschulte Pflegekraft rund um die Uhr zu Hause vor Ort. Diese Pflegekraft kommt sowohl für die Grundpflege des Menschen mit Demenz, als auch für sonstige hauswirtschaftliche Aufgaben auf. Voraussetzung dafür ist allerdings ein eigenes Zimmer für die Pflegekraft. Eine vollständige Kostendeckung durch die Pflegeversicherung ist dabei im Regelfall nicht möglich, so dass eigene Zuzahlungen notwendig werden.

Welche Wohn- und Versorgungsangebote außerhalb des eigenen Zuhauses gibt es?

Es gibt verschiedene Wohnmöglichkeiten für Menschen mit Demenz. Bei der Suche nach einer geeigneten Einrichtung sollte neben den persönlichen Wünschen stets auch der Schweregrad der Demenz beachtet werden.

Betreutes Wohnen in einem eigenen Appartement

Die Idee des „betreuten Wohnens“ ist, die Selbstständigkeit älterer Menschen so lange wie möglich zu erhalten. Je nach Bedarf können verschiedene Leistungen in Anspruch genommen werden.

Einige Einrichtungen des betreuten Wohnens befinden sich dabei auf dem Gelände von Pflegeeinrichtungen. Bei vorschreitender Pflegebedürftigkeit ist somit ein Umzug in eine Pflegeeinrichtung nicht unbedingt notwendig.

Zu den Grundleistungen des betreuten Wohnens gehören neben einer eigenen barrierefreien Wohnung ein Notruf-System, Gemeinschaftsräume, ein Hausmeisterservice und die Reinigung der Wohnung. Zusätzlich können auf Wunsch weitere Leistungen in Anspruch genommen werden. Dazu gehören zum Beispiel Leistungen in der ambulanten Pflege, Lieferung von Mahlzeiten, Erledigungen von Behördengängen, Fahrdienste und Begleitdienste oder bestimmte hauswirtschaftliche Leistungen wie Waschen oder Einkaufen. Die Kosten für das betreute Wohnen sind je nach Angebot sehr unterschiedlich.

Wohngemeinschaften für Menschen mit Demenz

In sogenannten Wohngemeinschaften für Menschen mit Demenz teilen sich etwa sechs bis zwölf Menschen mit Demenz eine gemeinsame Wohnung. Jeder Bewohner hat ein eigenes Zimmer zur Verfügung. Die Küche, das Wohnzimmer und das Bad werden von allen Bewohnern gemeinsam genutzt. Ausgebildete Pflegekräfte kümmern sich um die Bewohner. Dabei wird versucht, die Selbstständigkeit und Eigenverantwortlichkeit der Bewohner zu unterstützen.

Pflegeeinrichtungen

Anders als beim betreuten Wohnen stellen Pflegeeinrichtungen keine eigene Wohnung, sondern nur ein eigenes Zimmer zur Verfügung. Viele Pflegeeinrichtungen haben mittlerweile Versorgungsangebote, die speziell auf Menschen mit Demenz zugeschnitten sind. Welche besonderen Angebote die jeweilige Einrichtung für Menschen mit Demenz bietet, sollte vorher abgeklärt werden.

Tagespflegestätten

Es gibt spezielle Tagespflegestätten für ältere Menschen mit Demenz, die sowohl körperliche als auch geistige Unterstützung und Förderung bieten. Dabei kann die Tagespflegestätte mehrmals pro Woche besucht werden. Die Gestaltung des Tages wird dabei an die Wünsche und Fähigkeiten der Besucher angepasst. Ausgebildete Mitarbeiter begleiten und helfen bei gemeinsamen Aktivitäten wie Kochen, Backen, Singen oder der Gartenarbeit. Es gibt die Möglichkeit einen Probetag in der jeweilige Tagespflegestätte zu vereinbaren.

Wenn man weiter in seinem eigenen Zuhause wohnen möchte, dann sind diese Tagesstätten eine gute Möglichkeit. So kann es sehr förderlich sein, die eigenen vier Wände von Zeit zu Zeit zu verlassen. Kontakt mit anderen Menschen mit Demenz und regelmäßige Förderung können im Umgang mit der Demenz helfen. Auch pflegende Angehörige, die möglicherweise einen Beruf ausüben, können dadurch zeitweise entlastet werden. Pflegeversicherungen übernehmen je nach Pflegegrad einen Teil der Kosten. Mit einem finanziellen Eigenanteil muss aber stets gerechnet werden.

Welche weiteren Unterstützungsangebote gibt es?

Es gibt zusätzliche Unterstützungsangebote, die jedoch zeitlich begrenzt sind. Es kommt vor, dass pflegende Angehörige verhindert sind. Beispielsweise wenn die Angehörigen krank sind, in den Urlaub fahren wollen oder Zeit für eigene alltägliche Erledigungen brauchen. In diesem Fall gibt es einen Anspruch auf Unterstützung, um eine ausreichende Betreuung des Menschen mit Demenz sicherzustellen. Dann übernehmen speziell ausgebildete Pflegekräfte die Versorgung zu Hause. Manchmal kann die Versorgung zu Hause nicht ausreichend sein. Dann besteht ein Anspruch auf einen Aufenthalt in einer Pflegeeinrichtung in dieser Zeit.

Diese Unterstützungsmöglichkeiten nennt man Verhinderungspflege beziehungsweise Kurzeitpflege. Die Kosten werden von der Krankenkasse oder der Pflegekasse übernommen.

Wohnen und Versorgung – Das Wichtigste in Kürze

- Menschen mit Demenz können häufig auch bin an ihr Lebensende in ihrem eigenen Zuhause wohnen bleiben.

- Die Versorgung und Pflege im eigenen Zuhause kann von Angehörigen übernommen werden.

- Es gibt viele Unterstützungsangebote, um das Leben in den eigenen vier Wänden zu erleichtern (zum Beispiel ambulante Pflegedienste, 24-Stunden Pflegedienste).

- In einigen Fällen kann es jedoch mit fortschreitender Demenz schwierig werden, eine ausreichende Versorgung in den eigenen vier Wänden zu gewährleisten. In solchen Fällen kann eine andere Wohn- und Versorgungssituation sinnvoll sein.

- Es gibt viele unterschiedliche Wohnformen außerhalb des eigenen Zuhauses, die in Frage kommen könnten (zum Beispiel betreutes Wohnen, Wohngemeinschaften für Menschen mit Demenz, Pflegeeinrichtungen).

Entscheidungsfindung – Wohnen und Versorgung

Im Folgenden sehen Sie eine Tabelle. Auf der linken Seite in **grün** lesen Sie, was Menschen bewegt, jetzt zu planen, wie sie später wohnen und versorgt werden. Auf der rechten Seite in **blau** lesen Sie, was Menschen bewegt, jetzt nicht zu planen, wie sie später wohnen und versorgt werden.

**Was bewegt Menschen, jetzt zu planen, wie sie später wohnen und versorgt werden?**	**Was bewegt Menschen, jetzt nicht zu planen, wie sie später wohnen und versorgt werden?**
Ich weiß, dass ich somit in der Zukunft gut versorgt bin und muss mir deswegen keine Gedanken machen.<br><br>Eine gute Versorgung zu Hause oder eine andere Wohnform kann bei fortschreitender Demenz meinen Bedürfnissen besser gerecht werden.<br><br>Ich entlaste mich und meine Angehörigen, indem ich rechtzeitig plane, wie ich später wohne und versorgt werde.	Ich erwarte, dass sich meine Familie um mich kümmert.<br><br>Ich möchte nicht, dass mich jemand anderes als meine Familie versorgt.<br><br>Meine Familie wird die richtige Entscheidung treffen, wie ich später wohne und versorgt werde.

Persönliche Ansichten von Menschen mit Demenz und deren Angehörigen

Im Folgenden lesen Sie Aussagen von Menschen, die sich mit dem Thema Wohnen und Versorgung auseinandergesetzt haben. Es handelt sich dabei um ganz persönliche Ansichten der jeweiligen Personen.

„Weil meine Demenz fortgeschritten ist, hatte ich Schwierigkeiten zu Hause zurechtzukommen. Da habe ich mich für das „betreute Wohnen“ entschieden. Hier weiß ich, dass ich gut versorgt bin.“

Betroffene

Ich kann mir nicht vorstellen später in einem Altenheim zu wohnen. Dort kann man bestimmt nicht einfach tun, was man will. Man ist dann ständig auf andere Leute angewiesen und weniger unabhängig.“

Betroffener

„Für meinen Vater war es wichtig zu Hause wohnen zu bleiben. Als Familie konnten wir jedoch alleine nicht mehr ausreichend für seine Pflege aufkommen. Der ambulante Pflegedienst war somit eine große Hilfe für uns.“

Angehöriger

„Meine Mutter wohnt seit 30 Jahren in ihrem Haus. Es wäre für sie eine zu große Umstellung wegzuziehen. Ich bin guter Dinge, dass wir auch ohne professionelle Hilfe in der Zukunft gut zurechtkommen werden.“

Angehörige

Ihr Gefühl – Wohnen und Versorgung

Was ist für Sie besonders wichtig? Überlegen Sie, was für Sie mehr zutrifft und kreuzen Sie es dementsprechend an.

Ich möchte selbst darüber entscheiden, wie ich später wohnen möchte.

voll und ganz	eher ja	teils/teils	eher nein	überhaupt nicht

Wenn ich plane, wie ich wohnen und versorgt werden möchte, dann vereinfacht das mein Leben und das meiner Angehörigen.

voll und ganz	eher ja	teils/teils	eher nein	überhaupt nicht

Es entlastet mich, wenn ich bereits jetzt weiß, wo und wie ich später wohne und versorgt werde.

voll und ganz	eher ja	teils/teils	eher nein	überhaupt nicht

Ihre Einstellung – Wohnen und Versorgung

Wenn Sie sich mit dem Thema Wohnen und Versorgung beschäftigt haben, haben Sie sich vielleicht schon eine Meinung gebildet. Bitte kreuzen Sie Ihre aktuelle Einstellung an.

Möchte ich jetzt planen, wie ich später wohne und versorgt werde?

voll und ganz	eher ja	teils/teils	eher nein	überhaupt nicht

Ihre Notizen – Wohnen und Versorgung

Schreiben Sie bitte in das untenstehende Feld Ihre Fragen, Anmerkungen, Bedenken und nächsten Schritte zum Thema Wohnen und Versorgung. Sie können dieses Textfeld auch freilassen.

Wohnen und Versorgung – Fragen an den Angehörigen

Wenn Sie sich mit dem Thema Wohnen und Versorgung beschäftigt haben, haben Sie sich vielleicht schon eine Meinung gebildet. Bitte kreuzen Sie Ihre aktuelle Einstellung an.

Soll Ihr Angehöriger jetzt planen, wie er später wohnt und versorgt wird?

voll und ganz	eher ja	teils/teils	eher nein	überhaupt nicht

Ihre Notizen – Wohnen und Versorgung

Schreiben Sie bitte in das untenstehende Feld Ihre Fragen, Anmerkungen, Bedenken und nächsten Schritte zum Thema Wohnen und Versorgung. Sie können dieses Textfeld auch freilassen.

Wohnen und Versorgung – Was kann ich tun und wie kann ich vorgehen?

Wo kann ich mich zu diesem Thema beraten lassen?

Beratung über professionelle Unterstützung zu Hause

Die Alzheimer-Gesellschaften vor Ort bieten eine umfassende Beratung rund um ambulante Pflegedienste an. Auch bei den meisten Pflegediensten kann man sich direkt beraten lassen.

Beratung über Pflegeeinrichtungen

Es gibt die Möglichkeit, sich vorab über Pflegeeinrichtungen beraten zu lassen. Viele Einrichtungen bieten dazu auch Informationsveranstaltungen und Besichtigungstermine an. Hier stellt sich die jeweilige Einrichtung mit ihrem Konzept und ihren Räumlichkeiten vor. So kann ein guter Eindruck gewonnen werden, ob die jeweilige Einrichtung den eigenen Erwartungen und Bedürfnissen entspricht. Es kann auch hilfreich sein in der Einrichtung das Gespräch mit anderen Menschen mit Demenz oder deren Angehörigen zu suchen. Die Erfahrungen können bei der Entscheidung für eine Pflegeeinrichtung helfen.

Wartelisten

In den meisten Gemeinden gibt es Pflegeeinrichtungen für Menschen mit Demenz. Diese Plätze sind jedoch begrenzt. Bei vielen Einrichtungen gibt es Wartelisten, um sich für einen Wohnplatz anzumelden. Wenn man sich für eine Einrichtung entschieden hat, dann sollte man sich zeitnah auf die entsprechende Warteliste setzen lassen.

Wohnen und Versorgung – Wichtige Anlaufstellen und Adressen

Deutsche Alzheimer Gesellschaft e.V. Selbsthilfe Demenz

Deutsche Alzheimer Gesellschaft e.V. Selbsthilfe Demenz

Friedrichstraße 236, 10969 Berlin

Telefon: 030/259 37 95-0

https://www.deutsche-alzheimer.de

→ Hier können Sie sich rund um das Thema Pflege und Demenz beraten lassen

Bundesministerium für Familien, Senioren, Frauen und Jugend

www.wegweiser-demenz.de/informationen/betreuung-und-pflege

→ Hier finden Sie Informationen rund um das Thema Wohnen, Versorgung und Pflege bei Demenz

Zentrum für Qualität in der Pflege

http://bdb.zqp.de/

→ Hier finden Sie Informationen rund um das Thema Pflege

Bayrisches Staatsministerium für Gesundheit und Pflege

https://www.stmgp.bayern.de/service/ansprechpartner-und-fachstellen/

→ Hier finden Sie einen Überblick der Fachstellen rund um Gesundheit und Pflege

Bundesarbeitsgemeinschaft Wohnungsanpassung mit Adressverzeichnis der Beratungsstellen (bundesweit)

www.wohnungsanpassung-bag.de

→ Hier finden Sie einen Überblick von Wohnberatungsstellen zum Thema selbstständiges Wohnen

Heimverzeichnis (bundesweit):

www.heimverzeichnis.de

→ Hier finden Sie einen Überblick über Altenheime, Pflegeeinrichtungen und Seniorenresidenzen mit Lebensqualität

JETZT!

Autofahren und Fahreignung

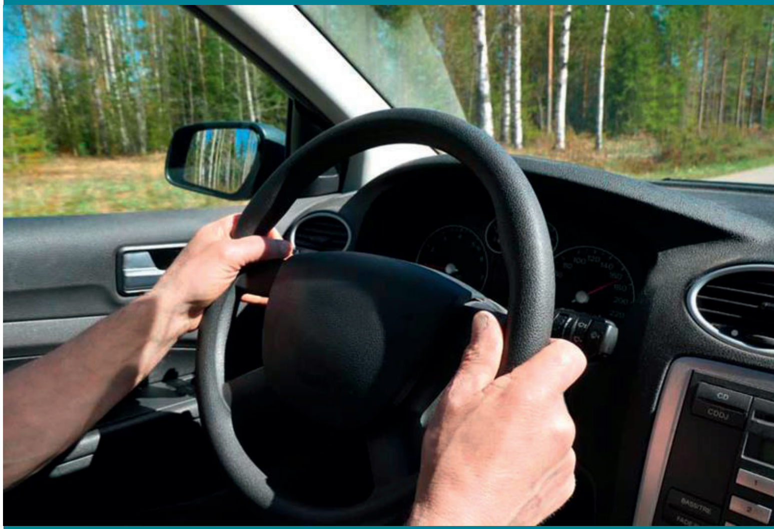

Thema: Autofahren und Fahreignung

ACHTUNG: Das Thema „Autofahren und Fahreignung“ unterscheidet sich deutlich von den vorherigen Themen. Jeder Autofahrer ist gesetzlich verpflichtet zu überprüfen, ob er noch sicher am Straßenverkehr teilnehmen kann. Wir möchten Sie deswegen dazu anregen, sich möglichst früh mit dem Thema Autofahren auseinanderzusetzten. Auch wenn Sie sich entscheiden, sich jetzt noch nicht mit dem Thema auseinander zu setzen, entbindet Sie das nicht von Ihrer gesetzlichen Pflicht.

→ **Möchte ich jetzt prüfen, ob ich noch sicher Auto fahren kann?**

Das Thema „Autofahren und Fahreignung“ bezieht sich auf alle motorisierten Kraftfahrzeuge. Dazu gehören neben dem Auto zum Beispiel Motorräder, Mopeds, Mofas.

Häufig gestellte Fragen – Autofahren und Fahreignung

Wie wirkt sich eine Demenz auf das Autofahren aus?

Statistiken zeigen, dass das Unfallrisiko bei Menschen mit einer Demenz erhöht ist. Bereits im Anfangsstadium einer Demenz kann die Fahrtauglicht beeinträchtigt sein. Diese Beeinträchtigungen nehmen im Verlauf der Demenz zu. Dabei gefährdet man nicht nur sich selbst, sondern auch seine Mitfahrer und andere Verkehrsteilnehmer. In den mittleren und späteren Stadien einer Demenz ist eine sichere Teilnahme am Straßenverkehr nicht mehr möglich.

Welche Pflichten habe ich als Autofahrer nach gesicherter Diagnosestellung einer Demenz und wie kann ich diesen Pflichten nachkommen?

Jeder Führerscheininhaber ist gesetzlich verpflichtet, eigenverantwortlich zu überprüfen, ob er noch sicher Auto fahren kann. Es gibt unterschiedliche Maßnahmen zur Überprüfung und Sicherstellung der Fahreignung:

<u>Auf Warnsignale achten</u>

Eine Demenz führt im Verlauf zu Einschränkungen von Fähigkeiten, die für das Autofahren notwendig sind. Schon kleine Fahrfehler können ein Hinweis darauf sein, dass sicheres Autofahren nicht mehr garantiert ist. Achten Sie deswegen bei sich auf folgende Warnsignale, wie zum Beispiel:

- Unabsichtliches Missachten von Verkehrsregeln
- Nicht angepasste Geschwindigkeit (zu langsam oder zu schnell)
- Schwierigkeiten, Verkehrszeichen zu verstehen
- Unfälle oder “Beinahe-Unfälle“
- Sich verfahren
- Beschädigungen am Auto
- Fahren in die falsche Richtung (zum Beispiel Einfahren in eine Einbahnstraße)
- Fahren auf der falschen Straßenseite
- Verwechseln von rechts und links
- Verwechseln von Gas und Bremse

<u>Soziales Umfeld miteinbeziehen</u>

Es ist nicht immer leicht, diese Warnsignale bei sich selbst zu erkennen. Bitten Sie deswegen Ihre Angehörigen, Freunde, Nachbarn und Mitfahrer, dass sie ebenfalls auf diese Warnsignale achten und Sie bei Auffälligkeiten darauf hinweisen.

Fahreignung prüfen lassen

Um sicherzustellen, ob Sie weiterhin am Straßenverkehr teilnehmen können, empfiehlt sich eine Abklärung Ihrer Fahreignung. Die hierfür unterschiedlichen Anlaufstellen finden Sie am Ende des Themas. Diese Kontrollen sollten regelmäßig wiederholt werden, um eindeutige Veränderungen in Ihrem Fahrverhalten frühzeitig zu erkennen.

Führerschein abgeben

Es besteht jederzeit die Möglichkeit, dass Sie Ihren Führerschein abgeben, wenn Sie selbst den Eindruck haben, nicht mehr sicher Auto fahren zu können. Der Führerschein kann bei der zuständigen Fahrerlaubnisbehörde abgegeben werden. Ihr Arzt oder professionelle Stellen zur Fahreignungsprüfung können diese Empfehlung ebenfalls aussprechen.

Maßnahmen zur Sicherstellung der Fahreignung

- Selbst auf Warnsignale achten
- Soziales Umfeld (Angehörige, Freunde, Nachbarn) auf Warnsignale achten lassen
- Fahreignung überprüfen lassen
- Führerschein abgeben

Mit welchen Konsequenzen muss ich rechnen, wenn ich trotz Fahruntauglichkeit weiter Auto fahre?

Fahruntauglich sein bedeutet, dass man nicht mehr sicher Auto fahren kann. Wenn man trotzdem weiter Auto fährt, stellt man für sich selbst und andere Verkehrsteilnehmer eine Gefahr dar. Sie und andere Personen können dadurch verletzt oder im schlimmsten Fall sogar getötet werden. Manche Menschen mit Demenz nutzen, trotz Warnungen ihres Arztes oder ihrer Angehörigen, weiterhin ihr Auto. Wenn mit einer erheblichen Gefährdung anderer Verkehrsteilnehmer zu rechnen ist, kann eine Begutachtung Ihrer Fahreignung in die Wege geleitet werden. Wenn eine eindeutige Fahruntauglichkeit vorliegt, wird Ihnen die Fahrerlaubnis entzogen und der Führerschein zwangsweise eingezogen.

Bei Menschen mit einer Demenz steht grundsätzlich die Haftpflichtversicherung für Schäden bei Verkehrsunfällen ein. Es kann jedoch zu einem Verlust des Versicherungsschutzes kommen, wenn der Unfall aufgrund der bereits bekannten Demenz passiert ist. In diesem Fall müssen Sie für entstandene Schäden selbst aufkommen.

Autofahren und Fahreignung – Das Wichtigste in Kürze

- Um sicher Auto zu fahren, braucht es eine Reihe an Fähigkeiten. Menschen mit einer Demenz verlieren diese Fähigkeiten im Verlauf ihrer Demenz, manchmal ohne es selbst zu merken.

- Als Führerscheininhaber sind Sie verpflichtet, eigenverantwortlich zu überprüfen, ob Sie noch sicher Auto fahren können.

- Achten Sie deswegen auf Warnsignale, die darauf hindeuten, dass sicheres Autofahren nicht mehr garantiert ist.

- Bitten Sie auch Ihre Angehörigen und Mitfahrer, auf Warnsignale zu achten und Sie auf Auffälligkeiten in Ihrem Fahrverhalten hinzuweisen.

- Lassen Sie ihre Fahreignung regelmäßig überprüfen.

- Im fortgeschrittenen Stadium einer Demenz, können Sie nicht mehr Auto fahren. Wenn Sie weiter Auto fahren, gefährden Sie dabei sich selbst, Ihre Mitfahrer und andere Verkehrsteilnehmer.

- Bei Fahruntauglichkeit und weiterer Teilnahme am Straßenverkehr müssen Sie bei von Ihnen verschuldeten Verkehrsunfällen mit rechtlichen und finanziellen Konsequenzen rechnen.

Entscheidungsfindung – Autofahren und Fahreignung

Im Folgenden sehen Sie eine Tabelle. Auf der linken Seite in **grün** lesen Sie, was Menschen bewegt, jetzt schon zu prüfen, ob sie noch sicher Auto fahren können. Auf der rechten Seite in **blau** lesen Sie, was Menschen bewegt, jetzt noch nicht zu prüfen, ob sie noch sicher Auto fahren können.

**Was bewegt Menschen, <u>jetzt zu prüfen</u>, ob sie noch sicher Auto fahren können?**	**Was bewegt Menschen, <u>jetzt noch nicht zu prüfen</u>, ob sie noch sicher Auto fahren können?**
Ich möchte sicherstellen, dass ich durch meine Fahrweise andere Verkehrsteilnehmer nicht gefährde.<br><br>Ich verhindere rechtliche und finanzielle Konsequenzen, zum Beispiel Probleme mit der Haftpflichtversicherung.<br><br>Meine Familie hat Veränderungen in meinem Fahrverhalten bemerkt.<br><br>Ich komme meiner gesetzlichen Vorsorgepflicht nach.	Ich muss mich erst damit auseinandersetzen, dass ich das selbständige Autofahren im Verlauf aufgeben muss.<br><br>Ich traue mir zu, dass ich selbst bemerke wenn ich nicht mehr sicher Auto fahren kann.<br><br>Der Gedanke, dass ich ohne Führerschein auf andere angewiesen sein werde, macht mir Sorgen.<br><br>Ich habe noch keine Alternative zum Autofahren gefunden.

Persönliche Ansichten von Menschen mit Demenz und deren Angehörigen

Im Folgenden lesen Sie Aussagen von Menschen, die sich mit dem Thema Autofahren und Fahreignung auseinandergesetzt haben. Es handelt sich dabei um ganz persönliche Ansichten der jeweiligen Personen.

„Neulich hätte ich fast ein Kind am Zebrastreifen übersehen. Da habe ich beschlossen, nicht mehr Auto zu fahren. Jetzt frage ich Angehörige oder Freunde, ob sie mich fahren können, oder ich nehme den Bus.“

Betroffener

„Ich fahre viel mit dem Auto. Autofahren bedeutet für mich, mobil und flexibel zu sein. Das ist mir sehr wichtig. Ich möchte nicht ständig von meiner Familie abhängig sein.“

Betroffene

„Mein Vater hat trotz unserer Einwände zuerst nicht einsehen können, dass er Schwierigkeiten mit dem Autofahren hat. Schlussendlich hat er aber dann doch erkannt, dass er nicht mehr Auto fahren sollte.“

Angehörige

„Meine Mutter ist demenzkrank und fährt trotzdem weiterhin Auto. In letzter Zeit sind mir Schrammen am Auto aufgefallen, die sich meine Mutter nicht erklären kann. Sie sagt, es sei alles in Ordnung.“

Angehöriger

Ihr Gefühl – Autofahren und Fahreignung

Was ist für Sie besonders wichtig? Überlegen Sie, was für Sie mehr zutrifft und kreuzen Sie es dementsprechend an.

Mir ist es wichtig, dass ich durch mein Autofahren andere Verkehrsteilnehmer nicht in Gefahr bringe.

voll und ganz	eher ja	teils/teils	eher nein	überhaupt nicht

Ich bin mir meiner Vorsorgepflicht bewusst, dass ich eigenverantwortlich überprüfen muss, ob ich noch fahrtauglich bin.

voll und ganz	eher ja	teils/teils	eher nein	überhaupt nicht

Ich gebe bei Fahruntauglichkeit meinen Führerschein lieber selber ab und verhindere, dass mir der Führerschein zwangsweise abgenommen wird.

voll und ganz	eher ja	teils/teils	eher nein	überhaupt nicht

Ihre Einstellung – Autofahren und Fahreignung

Wenn Sie sich mit dem Thema Autofahren und Fahreignung beschäftigt haben, haben Sie sich vielleicht schon eine Meinung gebildet. Bitte kreuzen Sie Ihre aktuelle Einstellung an.

Möchte ich jetzt prüfen, ob ich noch sicher Auto fahren kann?

voll und ganz	eher ja	teils/teils	eher nein	überhaupt nicht

Ihre Notizen – Autofahren und Fahreignung

Schreiben Sie bitte in das untenstehende Feld Ihre Fragen, Anmerkungen, Bedenken und nächsten Schritte zum Thema Autofahren und Fahreignung. Sie können dieses Textfeld auch freilassen.

Autofahren und Fahreignung – Frage an den Angehörigen

Wenn Sie sich mit dem Thema Autofahren und Fahreignung beschäftigt haben, haben Sie sich vielleicht schon eine Meinung gebildet. Bitte kreuzen Sie Ihre aktuelle Einstellung an.

Soll Ihr Angehöriger jetzt prüfen, ob er noch sicher Auto fahren kann?

voll und ganz	eher ja	teils/teils	eher nein	überhaupt nicht

Ihre Notizen – Autofahren und Fahreignung

Schreiben Sie bitte in das untenstehende Feld Ihre Fragen, Anmerkungen, Bedenken und nächsten Schritte zum Thema Autofahren und Fahreignung. Sie können dieses Textfeld auch freilassen.

Autofahren und Fahreignung – Was kann ich tun und wie kann ich vorgehen?

Welche Möglichkeiten habe ich, um meine Fahreignung zu prüfen?

Eine rechtsverbindliche Überprüfung der Fahreignung kann nur über die Fahrerlaubnisbehörde stattfinden. Die Behörde kann folgende Untersuchungen anordnen:

- Medizinisch-psychologische Untersuchung
- Gutachten eines Facharztes mit verkehrsmedizinischer Qualifikation

<u>Nicht rechtsverbindliche Überprüfung der Fahreignung</u>

Im Rahmen der Vorsorgepflicht kann man ebenfalls, ohne einen behördlichen Auftrag, eine Überprüfung der Fahreignung vornehmen lassen. Der Entzug der Fahrerlaubnis, auch bei negativem Ergebnis, ist hiermit nicht automatisch verbunden.

Auch ohne behördlichen Auftrag kann man ein Privatgutachten durch einen Facharzt mit verkehrsmedizinischer Qualifikation erstellen lassen. Einige zugelassene Begutachtungsstellen für Fahreignung, unter anderem der technische Überwachungsverein (TÜV), bieten sogenannte FitnessChecks für Senioren an. Eine andere Möglichkeit sind „Senioren-Trainings", die regelmäßig vom ADAC angeboten werden. Auch spezialisierte Fahrschulen bieten die Möglichkeit, die Fahrtüchtigkeit im Rahmen einer Beobachtungsfahrt mit einem Fahrlehrer überprüfen zu lassen.

Welche anderen Möglichkeiten zum Autofahren gibt es?

Der Verzicht auf das Autofahren bedeutet zunächst eine große Umstellung. Es gibt es viele Alternativen zum Autofahren:

- Fragen Sie Ihre Angehörigen, Freunde oder Nachbarn nach einer Mitfahrmöglichkeit.

- Nutzen Sie öffentliche Verkehrsmittel wie Bus oder Bahn. In vielen Städten gibt es spezielle Seniorentickets mit ermäßigtem Fahrpreis. Auch mit einem Schwerbehindertenausweis erhält man Vergünstigungen.

- Informieren Sie sich über sogenannte Seniorentaxis. Diese werden in vielen Gemeinden angeboten.

- Für viele Strecken benötigt es kein Auto. Seien Sie aktiv und gehen Sie zu Fuß. Damit tun Sie auch Ihrer Gesundheit einen Gefallen.

- Einkäufe müssen nicht unbedingt mit dem Auto erledigt werden. Viele Supermärkte bieten mittlerweile einen Lieferdienst an.

Autofahren und Fahreignung – Wichtige Anlaufstellen und Adressen

Sprechstunde Fahrtauglichkeit:

Klinik und Poliklinik für Psychiatrie und Psychotherapie (Sprechstunde Fahrtauglichkeit)

Nussbaumstraße 7, 80336 München

Tel: 089/4400-52774

Eine schriftliche Anmeldung durch einen Arzt oder Psychologen ist zwingend erforderlich

Begutachtungsstelle für Fahreignung München (DEKRA):

Bayerstr. 15, 80335 München

Tel: 089/5447938-0

TÜV SÜD Life Service GmbH:

Goethestraße 4, 80336 München

Telefon: 089 545428 3

→ Bei diesen Anlaufstellen können Sie ihre Fahreignung überprüfen lassen.

Es besteht außerdem die Möglichkeit bei Fahrschulen in Ihrem Umkreis, Ihre Fahreignung überprüfen zu lassen. Es empfiehlt sich, sich bei der jeweiligen Fahrschule vor Ort direkt zu informieren.

JETZT!

Weitere Vorausplanungen und Entscheidungen

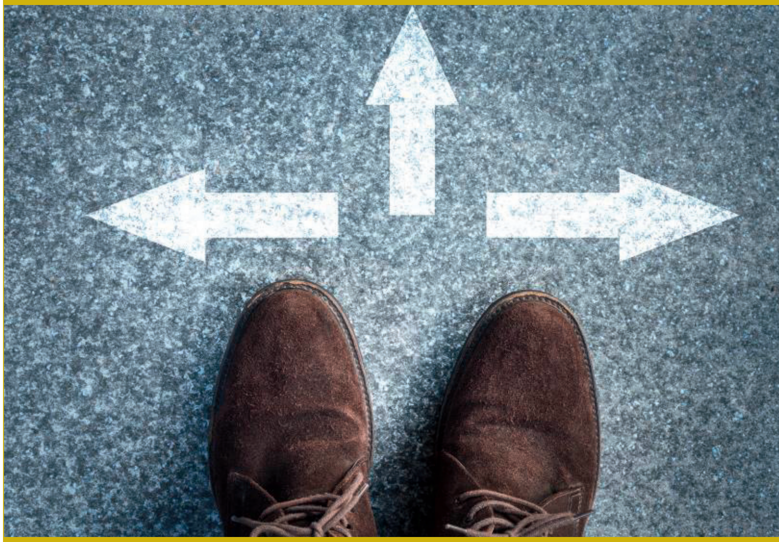

Weitere Vorausplanungen und Entscheidungen

Neben den bereits in der Orientierungs- und Entscheidungshilfe genannten Themen gibt es noch weitere Möglichkeiten der Vorausplanung und Entscheidungen, die bereits jetzt für die Zukunft getätigt werden können. Einige Themen sind schwierig und es fällt manchen Menschen nicht leicht, sich damit auseinanderzusetzen.

Versicherungen

Es lohnt sich, nach der Diagnose einer Demenz seinen Versicherungsschutz zu überprüfen.

Private Haftpflichtversicherung

Die private Haftpflichtversicherung zahlt zum Beispiel nur dann, wenn der Verursacher eines Schadens „deliktsfähig" ist. Man ist deliktsfähig, wenn man in der Lage ist zu erkennen, dass man einen Schaden verursacht hat. Dies ist im Stadium einer fortgeschrittenen Demenz häufig nicht mehr gegeben. Deswegen sollte bei einer privaten Haftpflichtversicherung eine „Deliktsunfähigkeit" mitversichert sein.

Rechtsschutzversicherung

Eine Rechtsschutzversicherung kann sinnvoll sein, wenn Sie zum Beispiel gegen sozialrechtliche Leistungsträger Klage einreichen wollen, zum Beispiel bei der Pflegeversicherung.

Mitgliedschaften in Vereinen und Verbänden

Sozialverband

Um eine kompetente Beratung und Vertretung in allen sozialrechtlichen Angelegenheiten zu erhalten, besteht die Möglichkeit, dass Sie in einem Sozialverband Mitglied (zum Beispiel Sozialverband VdK) werden. Der Mitgliedsbeitrag beläuft sich auf etwa 6 Euro im Monat.

Vereine

Mitgliedschaften in Vereinen, zum Beispiel Sportvereine oder Gesangsvereine, sind eine gute Möglichkeit mit anderen Personen Zeit zu verbringen und in Kontakt zu bleiben. Menschen mit Demenz können weiterhin vom Vereinsleben profitieren.

Weitere Informationen zum Thema Mitgliedschaften

Sozialverband VdK (https://www.vdk.de/deutschland)

Beratung zu Rentenversicherung, Krankenversicherung, Pflegeversicherung, Schwerbehinderung.

Schwerbehindertenausweis

Personen mit einer Demenz können ab einem bestimmten Schweregrad einen Schwerbehindertenausweis beantragen, der dem Inhaber einige Vorteile bringt:

- Wenn Sie noch berufstätig sind, haben Sie Anspruch auf zusätzliche Urlaubstage und einen erweiterten Kündigungsschutz. Sie können auch vorzeitig (frühestens mit 60 Jahren) ein flexibles Altersruhegeld beantragen.
- Wenn Sie zur Miete wohnen, haben Sie einen erhöhten Kündigungsschutz. In einigen Bundesländern gibt es auch für den behindertengerechten Umbau eine finanzielle Unterstützung.
- Des Weiteren gibt es je nach Grad der Schwerbehinderung Steuervergünstigungen für Betreuung und Pflege beziehungsweise für eine Haushaltshilfe und Preisnachlässe im öffentlichen Leben (zum Beispiel bei Bus und Bahn).

Wenn Sie einen Schwerbehindertenausweis beantragen wollen, wenden Sie sich an das Versorgungsamt. Formulare erhalten Sie beim Versorgungsamt, bei den Kommunen und bei Beratungsstellen für Behinderte. Holen Sie sich Hilfe, wenn Sie Probleme beim Ausfüllen des Antrages haben.
Meist dauert es mehrere Monate, bis Ihr Verfahren auf Antrag der Schwerbehinderung abgeschlossen ist. Es ist ratsam, sich hierbei von einer sozialrechtlichen Beratungsstelle (zum Beispiel beim Sozialverband VdK) unterstützen zu lassen.

Weitere Informationen zum Thema Schwerbehindertenausweis

Informationen finden Sie beim Bundesministerium für Familie, Senioren, Frauen und Jugend (https://www.wegweiser-demenz.de/startseite.html). Hilfe und Unterstützung beim Sozialverband VdK (https://www.vdk.de/deutschland).

Beruf und Rente

Wenn Sie noch berufstätig sind, sollten Sie sich die Frage stellen: „Wie lange will und kann ich weiterarbeiten?“
Wollen Sie weiterhin beruflich tätig sein, gibt es mehrere Möglichkeiten. Sie können zum Beispiel im selben Tätigkeitsfeld wie bisher weiterarbeiten oder Sie können das Tätigkeitsfeld wechseln. Oft ist es nicht leicht, eine Grenze zur Über- oder Unterforderung zu ziehen.
Unter bestimmten Voraussetzungen haben Sie Anspruch auf eine Erwerbsminderungsrente, wenn Sie in Folge Ihrer Erkrankung nicht mehr in der Lage sind, mehr als sechs Stunden am Tag zu arbeiten.
Informieren Sie sich gut über dieses Thema und lassen Sie sich sozialrechtlich beraten. Kompetente Beratung und sozialrechtliche Hilfe leistet zum Beispiel der Sozialverband *VdK.*

Weitere Informationen zum Thema Beruf und Rente

Ansprechpartner finden Sie beim Sozialverband VdK (https://www.vdk.de/deutschland) und bei der Deutschen Rentenversicherung (https://www.deutsche-rentenversicherung.de/Allgemein/de/Navigation/0_Home/home_node.html)

Nachlass

Viele Menschen kümmern sich bereits zu Lebzeiten um ihren Nachlass (Erbe). Dies können Sie mit einem Testament oder einem Erbvertrag regeln. Wenn Sie Ihren Nachlass nicht regeln, tritt automatisch die gesetzliche Erbfolge in Kraft, die in einer bestimmten Reihenfolge Ihre nächsten Angehörigen und/oder Ihren Ehepartner als Erben einsetzt.

Wenn Sie einen Erbvertrag abschließen müssen Sie zu diesem Zeitpunkt voll geschäftsfähig sein. Bei der Verfassung eines Testaments müssen Sie testierfähig sein. Testierfähig sein heißt, dass Sie den Inhalt des Testaments von sich aus bestimmt haben und keine weitere Person Einfluss gehabt hat. Um späteren Anfechtungen vorzubeugen, ist es ratsam, die Testier- beziehungsweise Geschäftsfähigkeit im Zweifelsfall von einem Arzt zu prüfen und bestätigen zu lassen.

Bei komplizierten Familienverhältnissen, mehreren Erben, großen Vermögenswerten oder zu vererbenden Unternehmen empfiehlt sich eine umfassende rechtliche Beratung.

Bestattung

Viele Menschen haben sehr klare Vorstellungen, wie sie sich ihre eigene Bestattung, die Todesanzeige und andere damit verbundene Dinge wünschen. Sie können etwa in einer Bestattungsverfügung festlegen, welche Personen vom Tod unterrichtet werden sollen, welche Beisetzungsart und welcher Beisetzungsort gewünscht werden, ob eine Trauerfeier oder ein Trauermahl ausgerichtet werden soll und welche Personen dazu eingeladen werden sollen.

Die Bestattungsverfügung sollte nicht Bestandteil des Testaments sein, da die Bestattung häufig schon vor der Testamentseröffnung erfolgt ist.

Forschungsverfügung für späte Stadien einer Demenz

Wenn Sie möchten, können Sie auch an Studien teilnehmen. Diese Studien testen zum Beispiel neue Behandlungen bei einer fortgeschrittenen Demenz. Es ist sehr wahrscheinlich, dass Sie selbst von dieser Forschung keinen Nutzen haben. Jedoch können die Ergebnisse der Studien später anderen Patienten mit einer Demenz helfen. Es kann sein, dass Sie nicht mehr einwilligungsfähig sind, wenn Sie für diese Studien in Frage kommen. Deshalb hat der Gesetzgeber große Hürden für diese Forschung aufgestellt:

- Sie müssen zuvor, solange Sie noch einwilligungs-fähig sind, schriftlich in die Teilnahme an der Forschung einwilligen (mit einer „Probandenverfügung“).

- Darin legen Sie fest, dass Sie an Studien teilnehmen wollen. Wann, ob und wie diese Studien stattfinden, ist zum Zeitpunkt der Einwilligung noch nicht klar.

- Sie müssen zuvor von einem Arzt umfassend über das Wesen, die Ziele, den Nutzen die Folgen, die Risiken und die Nachteile der Forschung aufgeklärt worden sein.

- Ihr Vorsorgebevollmächtigter oder Ihr Betreuer prüft, wenn es soweit ist, ob diese Festlegung auf Ihre aktuelle Situation zutrifft (wie bei den Festlegungen in einer Patientenverfügung).

- Wenn Sie aber zum Zeitpunkt der Forschung erklären oder in sonstiger Weise zum Ausdruck bringen, dass Sie nicht (mehr) teilnehmen wollen, so ist Ihr Wunsch zu respektieren.

Wenn Sie eine Forschungsverfügung erstellen wollen, kontaktieren Sie bitte universitäre Zentren der Demenzbehandlung, die meist auch Forschung durchführen.

Organspende und Gewebespende

In Deutschland muss jeder Mensch zustimmen, ob seine Organe gespendet werden dürfen oder nicht. Auf sogenannten Spenderausweisen können Sie notieren, ob Sie Organe oder anderes Gewebe nach Ihrem Tod spenden wollen. Sie legen dann auch fest, welche Organe oder Gewebe Sie spenden wollen. Wenn Sie selbst zu Lebzeiten keine Erklärung dazu abgegeben haben, werden im Todesfall Ihre nächsten Angehörigen gefragt, die in Ihrem Sinne entscheiden sollen. Deshalb sollten Sie mit Ihren Angehörigen über Ihre Einstellung dazu sprechen. Für die Organspende und Gewebespende gibt es keine grundsätzliche Altersgrenze, je nach Zustand der Organe können diese auch von älteren Menschen noch gespendet werden. Ob Organe für eine Spende geeignet sind, wird erst im Fall einer tatsächlichen Spende geprüft.

Sie können auch Gewebe wie Knochen, Zellen, Blutgefäße, Weichteile oder Augenhornhaut spenden. Teilweise werden daraus Arzneimittel hergestellt, die für die Behandlung verschiedener Erkrankungen bedeutsam sind.

Wenn sich Ihre Meinung bezüglich der Organspende ändern sollte, können Sie einen neuen Organspende-Ausweis ausfüllen und den alten Ausweis vernichten. Falls die Organspende Teil Ihrer Patientenverfügung ist, sollten Sie die Patientenverfügung diese dann dementsprechend abändern.

Sonderfall Gehirnspende

Es gibt die Möglichkeit nach dem Tod sein Gehirn und sein Rückenmark für Forschungszwecke zur Verfügung zu stellen und einen zentralen Dienst an der Gesellschaft leisten. Um Erkrankungen des Gehirns besser zu verstehen, wird die Bereitschaft und Mitarbeit von Personen mit diesen Erkrankungen sowie von deren Angehörigen, aber auch von "gesunden" Personen benötigt. Leider hilft diese Spende nicht mehr dem Spender selbst. Durch Forschung kann aber zukünftigen Patienten mit diesen Erkrankungen geholfen werden.

Weitere Informationen zum Thema Organspende und Gewebespende

Informationen zur Organspende und Gewebespende erhalten Sie zum Beispiel über Ihre Krankenkasse.

Sie können einen Organspende-Ausweis bei der Bundeszentrale für gesundheitliche Aufklärung (BZgA, 50819 Köln) bestellen. Telefonisch können Sie einen Organspende Ausweis beim Infotelefon Organspende unter der gebührenfreien Telefonnummer 0800-9040400 bestellen. Organspende Ausweise sind zudem in vielen Arztpraxen und Apotheken erhältlich.

Wenn Sie Ihr Gehirn spenden wollen, können Sie sich an die Neurobiobank der Ludwig-Maximilian-Universität in München wenden.
(http://www.neuropathologie.med.uni-muenchen.de/neurobiobank_muenchen/gewebespender/index.html).

Überblick über Ihren aktuellen Stand der Bearbeitung der verschiedenen Themen

Nachdem Sie sich nun ausführlich mit der Orientierungs- und Entscheidungshilfe beschäftigt haben, haben Sie in der untenstehenden Tabelle die Möglichkeit anzugeben, wie weit Sie bereits in der Entscheidungsfindung bei den einzelnen Themen gekommen sind. Kreuzen Sie an und tragen Sie das entsprechende Datum ein.

	**BEARBEITET**	**IN ARBEIT**	**NICHT BEARBEITET**
**VORSORGEVOLLMACHT**	☐ Datum:	☐ Datum:	☐ Datum:
**PATIENTENVERFÜGUNG**	☐ Datum:	☐ Datum:	☐ Datum:
**WOHNEN UND VERSORGUNG**	☐ Datum:	☐ Datum:	☐ Datum:
**AUTOFAHREN**	☐ Datum:	☐ Datum:	☐ Datum:

Hier haben Sie Platz für Ihre persönlichen Notizen

Hier haben Sie Platz für Ihre persönlichen Notizen

Hier haben Sie Platz für Ihre persönlichen Notizen

1. Version, München, Juli 2018

Mitwirkende

Diese Orientierungs- und Entscheidungshilfe wurde von einem Expertenteam der Technischen Universität und der Ludwig-Maximilians-Universität München verfasst. Bestehend aus:

- Prof. Dr. Johannes Hamann (Psychiater)
- Prof. Dr. Dr. Ralf Jox (Medizinethiker, Palliativmediziner, Neurologe)
- Dr. Katharina Bronner (Ärztin)
- Lea Bodner (Psychologin)

Des Weiteren beratend zur Seite standen:

- Dr. Ulrich Babinsky (Seelsorger)
- Tobias Bartschinski (Sozialpädagoge)
- Veronika Bernlochner (Sozialpädagogin)
- PD Dr. Alexander Brunnauer (Neuropsychologe)
- Prof. Dr. Janine Diehl-Schmid (Psychiaterin)
- Bernd Heise (Patientenvertreter)
- Angelika Mertin (Sozialpädagogin)
- Franziska Mettke (Ärztin)
- Prof. Dr. Robert Perneczky (Psychiater)
- Dr. Sabine Petri (Juristin)
- Prof. Dr. Jürgen in der Schmitten (Allgemeinmediziner)
- Helga Schneider-Schelte (Sozialpädagogin)
- Carola Sraier (Gesundheitswissenschaftlerin)
- Hans Steil (Palliativfachkraft)
- Renate Wiesent (Angehörigenvertreterin)

Die Beteiligten geben keine finanziellen Interessenskonflikte an.

Copyright:
Diese Version ist ausschließlich zur Verwendung für Teilnehmer im Rahmen des DGF-geförderten Projektes JETZT und den darin stattfindenden Beratungsgesprächen gedacht.

Hinweis:
Aus Gründen der besseren Lesbarkeit wird in der Orientierungs- und Entscheidungshilfe jeweils nur die männliche Form verwendet, die weibliche Form ist dabei jeweils eingeschlossen.

Klinikum rechts der Isar
Technische Universität München

Klinik und Poliklinik für Psychiatrie und Psychotherapie
Ismaninger Straße 22
81675 München
www.mri.tum.de

LUDWIG-MAXIMILIANS-UNIVERSITÄT MÜNCHEN

Ludwig-Maximilians-Universität München

Institut für Ethik, Geschichte und Theorie der Medizin
Lessingstraße 2
80336 München
www.uni-muenchen.de
